# Membrane mechanics dictate axonal pearls-on-a-string morphology and function

**DOI:** 10.1038/s41593-024-01813-1

**Published:** 2024-12-02

**Authors:** Jacqueline M. Griswold, Mayte Bonilla-Quintana, Renee Pepper, Christopher T. Lee, Sumana Raychaudhuri, Siyi Ma, Quan Gan, Sarah Syed, Cuncheng Zhu, Miriam Bell, Mitsuo Suga, Yuuki Yamaguchi, Ronan Chéreau, U. Valentin Nägerl, Graham Knott, Padmini Rangamani, Shigeki Watanabe

**Affiliations:** 1 Department of Cell Biology, Johns Hopkins University School of Medicine, Baltimore, MD USA; 2 Department of Mechanical and Aerospace Engineering, Jacobs School of Engineering, University of California, San Diego, La Jolla, CA USA; 3 Neurobiology Course, The Marine Biological Laboratory, Woods Hole, MA USA; 4 Neuroscience Graduate Program, McMaster University, Hamilton, Ontario Canada; 5 Application Department, EPBU, JEOL Company, Ltd., Tokyo, Japan; 6 Bordeaux Neurocampus, Université de Bordeaux, Bordeaux, France; 7 Interdisciplinary Institute for Neuroscience, UMR 5297, Centre National de la Recherche Scientifique, Bordeaux, France; 8 Bioelectron Microscopy Core Facility, École Polytechnique Fédérale de Lausanne, Lausanne, Switzerland; 9 Department of Pharmacology, School of Medicine, University of California, San Diego, La Jolla, CA USA; 10 Solomon H. Snyder Department of Neuroscience, Johns Hopkins University School of Medicine, Baltimore, MD USA; 11 Present Address: Department of Molecular Biology, University of California, San Diego, La Jolla, CA USA; 12 Present Address: Department of Basic Neurosciences, Geneva University Neurocenter, Faculty of Medicine, University of Geneva, Geneva, Switzerland; 13 Present Address: Universitätsmedizin Göttingen, Georg-August-Universität, Zentrum Anatomie, Göttingen, Germany

**Keywords:** Cellular neuroscience, Membrane biophysics, Action potential generation

## Abstract

Axons are ultrathin membrane cables that are specialized for the conduction of action potentials. Although their diameter is variable along their length, how their morphology is determined is unclear. Here, we demonstrate that unmyelinated axons of the mouse central nervous system have nonsynaptic, nanoscopic varicosities ~200 nm in diameter repeatedly along their length interspersed with a thin cable ~60 nm in diameter like pearls-on-a-string. In silico modeling suggests that this axon nanopearling can be explained by membrane mechanical properties. Treatments disrupting membrane properties, such as hyper- or hypotonic solutions, cholesterol removal and nonmuscle myosin II inhibition, alter axon nanopearling, confirming the role of membrane mechanics in determining axon morphology. Furthermore, neuronal activity modulates plasma membrane cholesterol concentration, leading to changes in axon nanopearls and causing slowing of action potential conduction velocity. These data reveal that biophysical forces dictate axon morphology and function, and modulation of membrane mechanics likely underlies unmyelinated axonal plasticity.

## Abstract (web summary)

Axons have always been assumed to be cylindrical. Using in silico modeling and cryopreservation of tissues, Griswold et al. demonstrate that unmyelinated axons of the mammalian central nervous system exhibit pearls-on-a-string morphology through their entire length.

## Main

Axons are ultrathin membrane tubes specialized for rapid conduction of action potentials (APs) to convey information, even across a tissue or whole organism. It is well appreciated that AP propagation is finely tuned by the intricate morphology of axons^1–5^ and their cable-like properties^6,7^. During AP conduction in large-diameter axons, axonal diameter increases to lower axial electrical resistance^4,8–11^. In the mammalian central nervous system, high-frequency electrical stimulation (HFS) induces nanoscale remodeling of axonal morphology, where a transient enlargement of synaptic varicosities (by 20%) is followed by a sustained widening of the axons (by 5%)^12^. This, in turn, leads to bidirectional changes in AP conduction velocity, as predicted by the cable theory considering the biophysical effects of membrane capacitance and axial resistance. Thus, seemingly minute changes in axon morphology can sensitively tune AP conduction and overall neuronal function.

The morphology of small-diameter membrane tubes like axons is dictated by the biophysical properties of the membrane and underlying cytoskeleton. Electron microscopy (EM) reconstruction of unmyelinated rat hippocampal axons from fixed acute slices shows that their diameter is, on average, only 170 ± 40 nm (ref. ^13^). Such small-diameter biological membrane tubes under physiological conditions should be susceptible to forming what is known in the field of biophysics as a pearls-on-a-string morphology. This morphology arises due to pearling instability in the presence of tension, like in vitro membrane tubes of similar diameters^14–21^.

Regarding the factors governing axonal morphology, pearling behavior is noted in large (2.5 µm in diameter) unmyelinated axons when factors such as osmotic pressure^20^ and tension^22^ are modulated. Likewise, unmyelinated axons also exhibit an extreme form of pearling, which in the neurodegeneration field is also called beading, as they degenerate, presumably due to the increase in membrane tension and the loss of cytoskeleton integrity^23^. These data suggest that axons behave in a manner consistent with membrane pearling instability. However, analysis of axon morphology is classically performed after tissue fixation. In these studies, most unmyelinated axons appear relatively uniform in diameter by light microscopy and EM experiments^13^. Thus, it is unclear how much biophysical properties of membranes alone contribute to the shape of small-diameter unmyelinated axons in the mammalian central nervous system under physiological conditions.

Beyond the membrane, the contribution of the cytoskeleton to axon morphology has become more appreciated after the discovery of the membrane periodic cytoskeleton (MPS). Unlike other cellular compartments, axons have a unique cytoskeletal structure^24–28^, in addition to more traditional cortical actin^25,27^. The MPS is composed of actin rings 190 nm apart with scaffolding spectrin tetramers in between. Many other proteins, such as ankyrin G^24^ and voltage-gated sodium channels (NaVs)^25^, associate with this structure and are thus also periodically spaced. However, the MPS and its relationship to the membrane remains unclear. Studies on membrane tension suggest that the axonal plasma membrane is not as tightly linked to the underlying cytoskeleton but rather has a loose anchoring, unlike in other traditional cells^29^. Further studies examining axon resilience to stretch show that the MPS, specifically actin and spectrin, buffer overall axon strain, but how the membrane that overlies the MPS reacts during such stretching remains unclear^30^.

Here, we used high-pressure freezing EM and in silico modeling to determine the ultrastructure of axon morphology in mouse neurons and the contribution of membrane mechanics to the morphology. High-pressure freezing circumvents fixation artifacts like membrane distortion and protein aggregation and preserves membrane morphology in a near-native state^31–33^. We find that axons are not simple cylindrical tubes but rather exhibit nanoscopic pearls-on-a-string morphology due to minimization of the Helfrich–Canham energy (including bending, tension and osmotic pressure terms). Manipulation of biophysical factors changes the conduction velocity of APs. Thus, our study provides deeper insight into how axon morphology and function are controlled by a delicate balance of biophysical forces acting on the plasma membrane.

## Results

### Axons are pearled under physiological conditions

Membrane tubes in vitro typically form pearling due to tension-driven instability^15^. Although ultrathin membrane tubes like unmyelinated axons are susceptible to such biophysical changes, previous work has assumed that axons are tubular^13,34–38^. However, ultrastructural analysis is typically performed on samples prepared with aldehyde-based fixatives under conditions that do not preserve fine morphology. Thus, to interrogate the morphology of unmyelinated axons, we performed high-pressure freezing and EM analysis of neurons from acutely extracted mouse brain tissue (postnatal days 42–70 (P42–70), as per Tamada et al.^39^), organotypic slice culture and dissociated hippocampal culture from embryonic day 18 (E18) mouse pups. These three types of tissue preparations were chosen because they have different biophysical environments and may display distinct morphologies^40^.

**Fig. 1:**
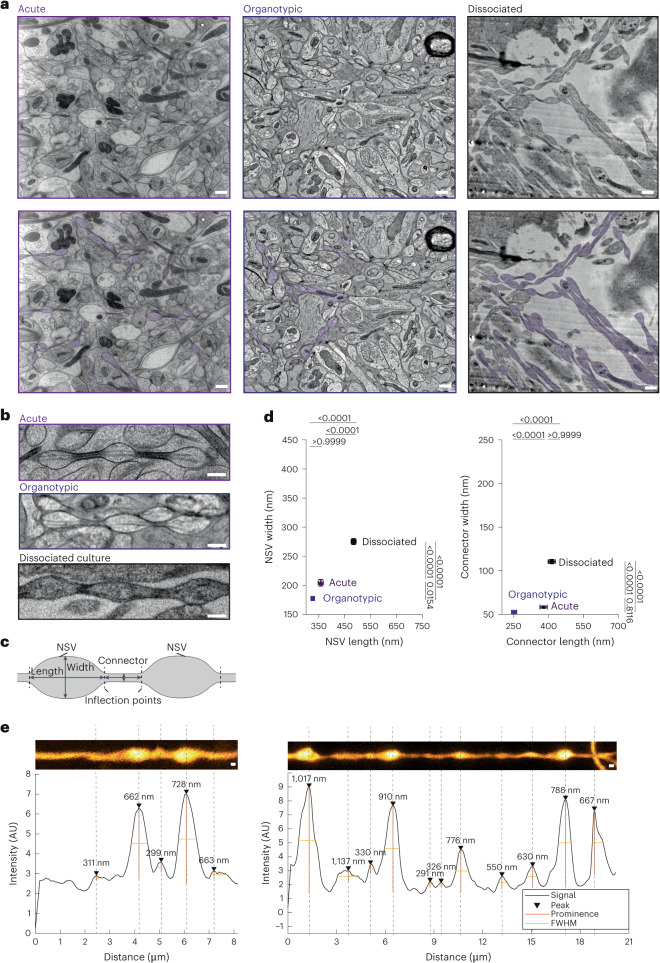
Axons are pearled, not tubular, under homeostatic conditions. **a**, Representative electron micrographs from acutely extracted mouse brain tissue (left), organotypic slice cultures of mouse hippocampus (middle) and dissociated mouse hippocampal neuronal culture after high-pressure freezing. Some axons in each micrograph are traced and color coded on the bottom; scale bars, 500 nm. **b**, High-magnification images of axons representative of each condition. More example micrographs are found in Extended Data Fig. 1a,b,e; scale bars, 200 nm. **c**, A schematic showing two NSVs flanked by a connector. Inflection points define the boundary between these two features. Both width and length are measured at NSVs and connectors, as shown. **d**, Plots showing dimensions of NSVs (left) and connectors (right) from indicated tissue types. Dimensions are measured from three independent samples for acutely extracted brain tissue and dissociated neuron culture and one for organotypic slices; *n* = 30 axons from each acutely extracted sample, *n* = 133 axons from the organotypic sample, and *n* = 100 axons from each dissociated sample. Super plots showing variability are available in Extended Data Fig. 1f. Data are shown as mean ± s.e.m. and were analyzed by Kruskal–Wallis test, followed by a Dunn’s multiple comparison test. **e**, Representative STED micrographs showing axons from organotypic slice cultures of mouse hippocampi. The numbers represent the length of each NSV, measured at full-width half-maximum (FWHM) using MATLAB scripts. Numbers indicate the measured length at each NSV; scale bars, 200 nm; AU, arbitrary units. Source data

**Extended Data Fig. 1:**
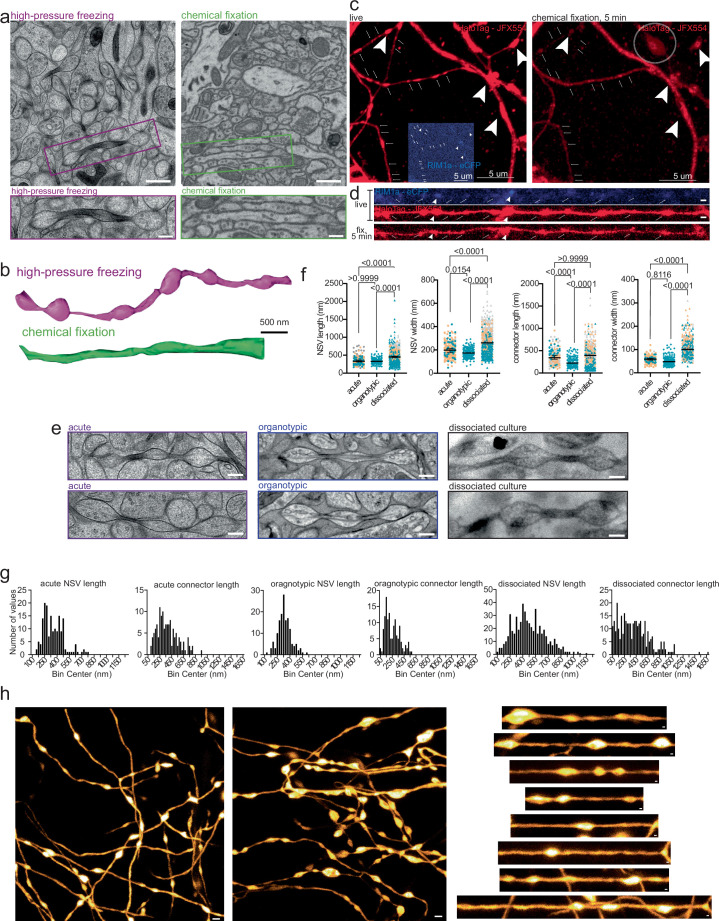
Pearled morphology is observed for all axons except after chemical fixation. **a**. Example micrographs showing acutely extracted mouse brain tissue either high-pressure frozen (left) or chemically fixed (right). The boxed axons are reconstructed using IMOD. Note that chemically fixed axons are like cylindrical tubes. Scale bar: 500 nm. **b**. Higher magnification images of the boxed area in a. Scale bar: 200 nm. **c**. AiryScan images of axons live without (left) or after 5 min of fixation (right). Inset shows synapses marked by RIM1a-CFP. Arrowheads mark synapses, arrows mark NSVs, circle marks extreme axon pearling associated with degenerating axons. Scale bar: 5 µm. **d**. Further images of an axon from a separate culture live without (middle) or after 5 min of fixation (bottom). Synapses marked by RIM1a-CFP (top). Arrowheads mark synapses, arrows mark NSVs. Scale bar: 1 µm. **e**. Additional representative images of axons from acutely extracted brain tissue, organotypic slice culture, and dissociated neuron culture. Scale bar: 200 nm. **f**. Super Plots of Fig. 1d, showing experimental variability. Median and 95% confidence intervals are shown. N = 3 and n = 30 axons from each acutely extracted sample, n = 133 axons from the organotypic sample, N = 3 and n = 100 axons from each dissociated sample. Kruskal-Wallis test, followed by Dunn’s multiple comparison test was used. Each color represents one replicate. Each dot is one axon. **g**. Frequency distributions for acute, organotypic, and dissociated culture of either NSV lengths or connector lengths. **h**. Additional STED micrographs showing pearled axon morphology in live neurons from organotypic slice cultures. Scale bar: 500 nm. Individual axons are straightened and represented on the right panels. Scale bar: 200 nm. Source data

As in previous studies^13,34,41^, axons appeared cylindrical in chemically fixed tissues (Extended Data Fig. 1a,b). By contrast, when prepared using the high-pressure freezing method, axons in all preparation types exhibited a pearls-on-a-string morphology (Fig. 1a,b and Extended Data Fig. 1a–d), with changes in dimension depending on culturing conditions. Pearled regions are defined as nonsynaptic varicosities (NSVs), and the region between two NSVs is designated as the connector with boundaries defined by the inflection points (Fig. 1b,c). To quantify this morphology, the widths and lengths of NSVs and connectors were measured. The axonal morphology of acute slices and organotypic slices was similar (Fig. 1d and Extended Data Fig. 1e–g). Cultured neurons were slightly larger in all dimensions (Fig. 1d, Table 1 and Extended Data Fig. 1f,g), most likely reflecting the changes in biophysical properties associated with substrate stiffness^40^. Nonetheless, these results suggest that unmyelinated axons indeed exhibit membrane pearling. Hereafter, these structures are referred to as nanopearling to distinguish them from macroscale membrane enlargements occurring in degenerating axons.

**Table 1:** Average dimensions and s.e.m. of axon NSVs and connectors

Sample name		Sample type	Treatment	NSV length (nm)	NSV width (nm)	Connector length (nm)	Connector width (nm)
Organotypic		Organotypic		326 ± 7	177 ± 3	252 ± 10	52 ± 2
Acute		Acute slice		357 ± 10	204 ± 6	380 ± 15	58 ± 1
Dissociated		2D		600 ± 9	311 ± 5	552 ± 14	127 ± 3
Chemical treatments	0.2% DMSO	2D	DMSO, 30 min	630 ± 13	318 ± 5	603 ± 18	136 ± 3
		2D	DMSO, 1 h	632 ± 18	323 ± 7	584 ± 20	153 ± 5
	0.1% DMSO	2D	DMSO, 1 h	602 ± 12	351 ± 7	518 ± 19	115 ± 3
	LatA	2D	LatA, 30 min	625 ± 11	323 ± 5	574 ± 20	136 ± 4
		2D	LatA, 1 h	584 ± 21	326 ± 11	588 ± 18	123 ± 6
	CytoD	2D	CytoD, 1 h	640 ± 17	342 ± 9	611 ± 19	134 ± 4
	Blebbistatin	2D	Blebbistatin, 1 h	590 ± 11	295 ± 4	431 ± 12	215 ± 5
	Nocodazole	2D	Nocodazole, 1 h	587 ± 10	297 ± 3	446 ± 13	195 ± 6
	MβCD	2D	MβCD 5 mM	550 ± 17	312 ± 8	540 ± 20	116 ± 4
	Control	2D	No treatment	640 ± 17	310 ± 7	490 ± 15	136 ± 4
*Sptbn1* KD		2D	Scb	728 ± 15	363 ± 6	633 ± 19	178 ± 6
		2D	*Sptbn1* KD	651 ± 14	335 ± 6	585 ± 19	138 ± 5
Osmolarity		2D	300 mOsm	480 ± 9	275 ± 5	410 ± 15	111 ± 2
		2D	600 mOsm	370 ± 8	190 ± 4	430 ± 16	106 ± 3
		2D	150 mOsm	610 ± 13	390 ± 8	661 ± 21	107 ± 3
		2D	300 mOsm	510 ± 10	250 ± 4	490 ± 19	114 ± 3
		2D	280 mOsm	565 ± 10	268 ± 4	540 ± 16	125 ± 3
		2D	400 mOsm	472 ± 8	228 ± 3	560 ± 13	118 ± 3
Stimulation	No stim	2D		449 ± 12	232 ± 5	462 ± 15	118 ± 3
	5 min	2D		541 ± 15	262 ± 6	512 ± 18	135 ± 4
	30 min	2D		526 ± 14	287 ± 7	510 ± 18	118 ± 4
	MβCD no stim	2D	MβCD 5 mM	405 ± 10	242 ± 6	421 ± 15	97 ± 3
	MβCD 5 min	2D	MβCD 5 mM	477 ± 14	293 ± 9	463 ± 15	111 ± 3
	MβCD 30 min	2D	MβCD 5 mM	448 ± 13	278 ± 7	426 ± 16	108 ± 3

Abbreviations: 2D, two-dimensional; Scb, scramble; stim, stimulation.

To ensure that axon nanopearling was not induced by our experimental conditions, we performed live-cell superresolution imaging of organotypic mouse hippocampal slice cultures at 21–35 days in vitro (DIV 21–35) and dissociated hippocampal neuron cultures at DIV 21. Cytosolic green fluorescent protein (GFP) was expressed using Sindbis virus with stereotaxic injection in the CA3 area of hippocampal slices ~36 h before the experiments. Stimulated emission depletion (STED) microscopy analysis of live tissues showed the nanopearling behavior of axons in all axons observed, similar to the morphology observed by EM (Fig. 1e and Extended Data Fig. 1h). Live-cell imaging of cultured neurons expressing cytosolic HaloTag–JFX554 also showed nanopearling in all axons (Extended Data Fig. 1c,d). The nanopearls were stable over the duration of imaging; we did not observe movement nor coalescence of these structures. This suggests that the pearls are not the outcome of the transport of large organelles or bulky cargoes, which could deform the membrane due to their size. These regions lacked active zone protein RIM1, marked by coexpression of RIM1–enhanced cyan fluorescent protein (RIM1–eCFP; Extended Data Fig. 1c,d), indicating the nonsynaptic nature of nanopearls. Importantly, addition of 4% paraformaldehyde caused loss of nanopearls within 5 min, making axons more cylindrical (Extended Data Fig. 1c,d) as observed in EM studies (Extended Data Fig. 1a,b), and excessive pearling of axons (circled in Extended Data Fig. 1c), indicative of dying neurons^42,43^. These data suggest that nanopearling is a prominent feature of unmyelinated axons.

### Membrane mechanics regulate axon morphology

**Fig. 2:**
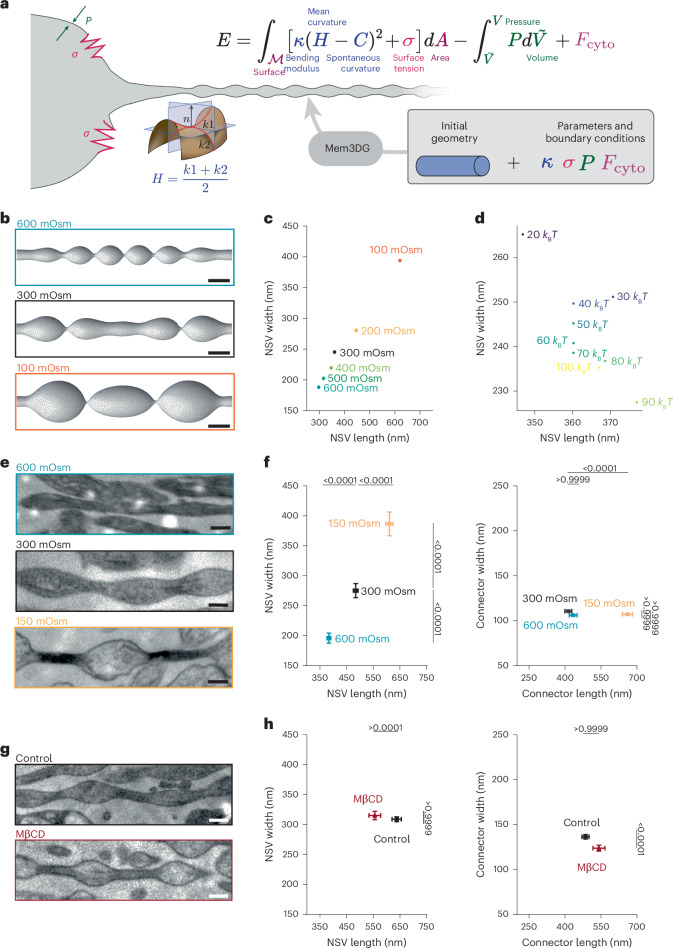
Membrane mechanics dictate axon nanopearling. **a**, Axon morphology is modeled using the classic Helfrich membrane model and governed by membrane bending, surface tension and osmotic conditions. **b**, Model prediction of axon morphology under the indicated osmotic conditions; scale bars, 200 nm. **c**, Plot showing the dimensions of NSVs at the indicated osmotic conditions. Note that NSV size is inversely scaled with the external osmotic pressure. **d**, Plot showing the dimensions of NSVs with varying membrane rigidity, ranging from 20 *k*_B_*T* to 100 *k*_B_*T*. **e**, Example micrographs of axons high-pressure frozen under the indicated osmotic conditions. More example micrographs are available in Extended Data Fig. 3a; scale bars, 200 nm. **f**, Plots showing the dimensions of NSVs (left) and connectors (right) from neurons in **e**; *n* = 100 axons from three replicates. Super plots showing variability are available in Extended Data Fig. 3b. Data are shown as mean ± s.e.m. and were analyzed by Kruskal–Wallis test, followed by a Dunn’s multiple comparison test. **g**, Example micrographs of axons from cultured neurons treated with sham (control) or 5 mM MβCD for 30 min. More example micrographs are available in Extended Data Fig. 4c; scale bars, 200 nm. **h**, Plots showing the dimensions of NSVs (left) and connectors (right) from neurons in **g**; *n* = 100 axons from three replicates. Super plots showing variability are available in Extended Data Fig. 4d. Data are shown as mean ± s.e.m. and were analyzed by Kruskal–Wallis test, followed by a Dunn’s multiple comparison test. Source data

We first considered whether the plasma membrane material properties could be driving the experimentally observed nanopearled shape. Building on the rich literature of membrane continuum mechanics, we constructed a model for predicting the membrane shapes of unmyelinated axons (Fig. 2a). We model the membrane region as a thin elastic surface with energy given by the equation displayed in Fig. 2a; this is the classic Helfrich Hamiltonian, which represents the elastic energy as a function of the surface curvatures^44,45^. A few parameters appear in the Hamiltonian, namely, the bending rigidity, spontaneous curvature, tension and pressure. Because we do not know the precise value or the specific isolated contributions to these parameters from the membrane, cortex and so on, we performed a parameter sweep over a range of values to mimic the total contributions of these elements. In this sense, the choice of the rigidity parameter should be interpreted as the net rigidity from all sources near the membrane surface and likewise for other terms. Minimizing the energy with respect to the shape yields solutions for mechanical equilibrium^46–48^. We used a computational approach, Mem3DG^49^, which represents the membrane using triangulated meshes and calculates the Helfrich energy and corresponding forces using strategies from discrete differential geometry.

**Extended Data Fig. 2:**
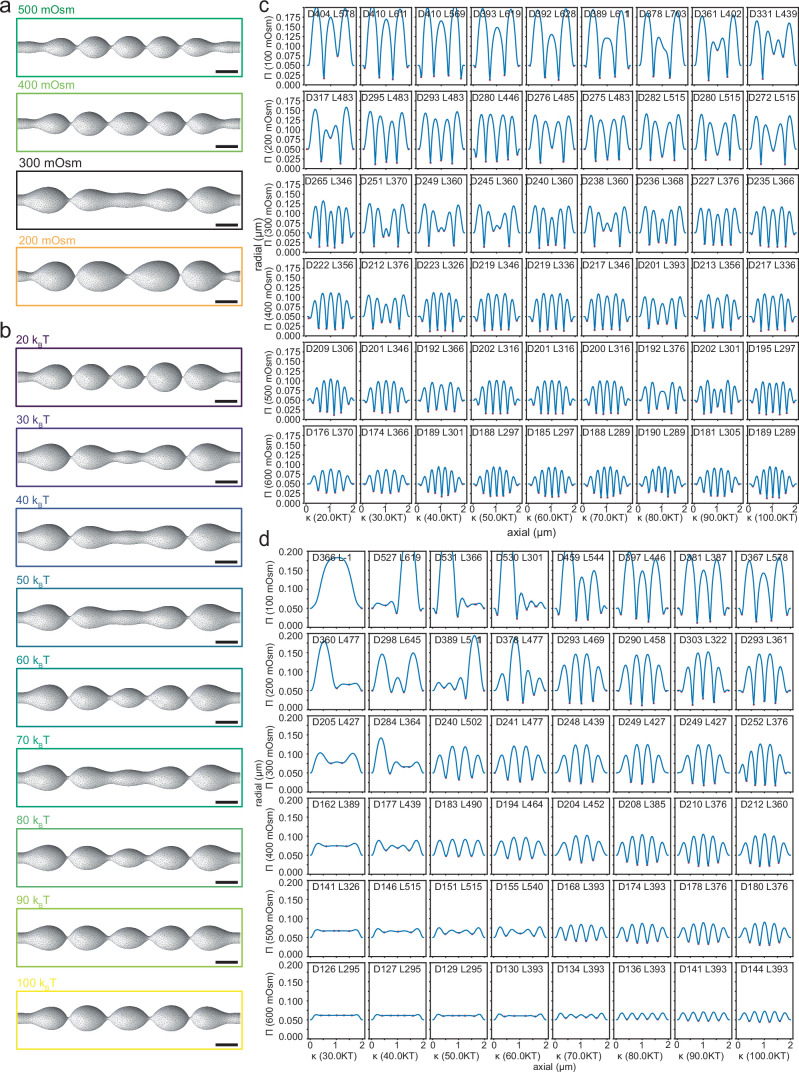
Manipulation of membrane mechanics predicts changes in axonal pearling. **a**. Axon morphology is modeled using classic Helfrich membrane model and governed by the membrane bending, surface tension, and osmotic condition. 3D model prediction of axon morphology in indicated osmotic conditions, tension of 0.001 mN/m, and bending rigidity of 50kBT. Scale bar: 200 nm. **b**. 3D model prediction of axon morphology at indicated bending rigidity, tension of 0.001 mN/m, and osmolarity 300 mOsm. Scale bar: 200 nm. **c**. Rotationally averaged shape profiles of axons under constant 0.001 mN/m tension and varying osmotic conditions and bending rigidity. The bead diameter (D) and length (L) in nanometers are given as a text inset for each condition. **d**. Rotationally averaged shape profiles of axons under constant 0.01 mN/m tension and varying osmotic conditions and bending rigidity. The bead diameter (D) and length (L) in nanometers are given as a text inset for each condition. Source data

Starting from an open-ended cylindrical tube connected to implicit membrane reservoirs, we varied the osmotic pressure, bending rigidity and membrane tension systematically and obtained the membrane geometries (Extended Data Fig. 2). Geometries for all conditions exhibited nanopearled morphologies with varying varicosity. Consistent with prior work on membrane tube mechanics^15,16,20,50–52^, manipulation of osmotic pressure on membranes has a strong influence on nanopearled morphology. Increasing solvent osmolarity reduces NSV width and length (Fig. 2b,c). Increasing tension also constrains the ability of the system to increase surface area, leading to reduced NSV width and length (Extended Data Fig. 2c,d), albeit this is a smaller effect than the osmotic pressure contribution (Extended Data Fig. 2). However, the bending rigidity had a modest effect on NSV geometry (Fig. 2d). With the assumption that model parameters such as spontaneous curvature, tension and bending rigidity are homogeneous, the output geometries are limited to periodic unduloid-like shapes^17,21^, and, thus, we could not capture the behavior of connectors, which would require the introduction of arbitrary heterogeneity^52^ or additional speculative physics. In summary, our simulations predict that NSVs, driven by a membrane pearling instability, scale inversely with osmotic conditions, where increasing the osmolarity of the milieu decreases both the NSV length and width.

**Extended Data Fig. 3:**
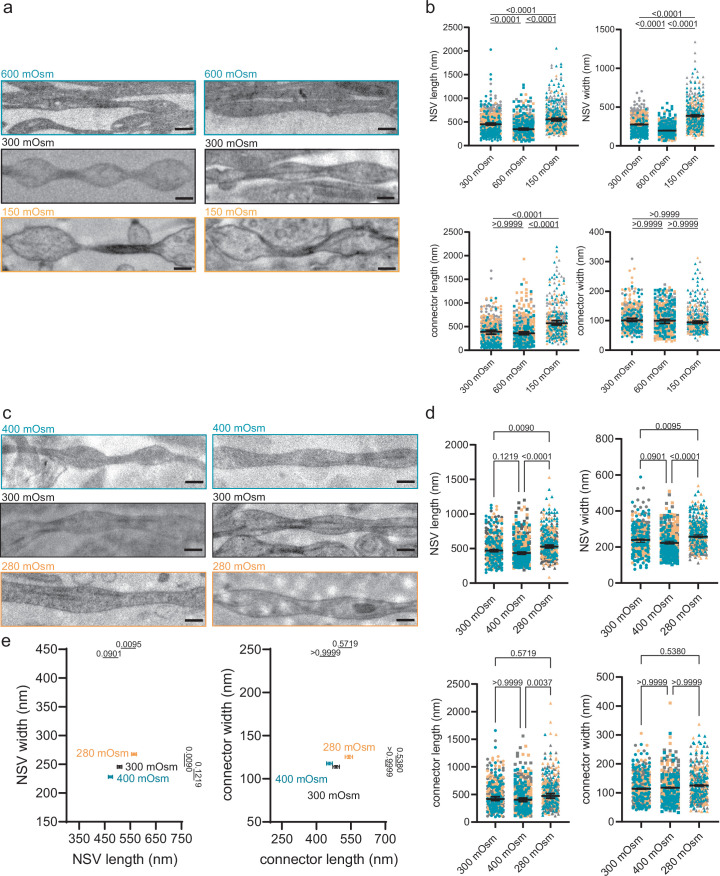
Manipulation of osmolarity causes changes in axonal pearling. **a**. Additional example micrographs of axons high-pressure frozen at indicated osmotic conditions. Scale bar: 200 nm. **b**. Super Plots of Fig. 2f, showing experimental variability. **c**. Example micrographs of axons high-pressure frozen at indicated osmotic conditions. Mannitol was used to adjust the osmolarity. Scale bar: 200 nm. **d**. Super Plots of Extended Data Fig. 2e, showing the dimensions of NSVs at indicated osmotic conditions. **e**. Plot showing the dimensions of NSVs at indicated osmotic conditions. Mean and SEM are plotted. N = 3 independent cultures, n = 100 axons each. In all super plots median and 95% confidence intervals are shown. N = 3 independent cultures and n = 100 axons each. Each color represents one replicate. Each dot is one axon. All data analyzed using Kruskal-Wallis test, followed by Dunn’s multiple comparison test.

To test the modeling predictions, ultrastructural analysis was performed on high-pressure frozen dissociated hippocampal culture (DIV 21) while manipulating the osmotic pressure from isotonic conditions to either hyper- (600 mOsm) or hypotonic (150 mOsm) conditions (Fig. 2e,f). In general, a hyperosmotic solution would shrink membranes, resulting in a reduction in membrane tension, whereas a hypo-osmotic solution would do the opposite. As predicted by the model (Fig. 2b,c), the dimensions of NSVs were inversely correlated with the osmolarity (Fig. 2e,f and Extended Data Fig. 3a,b). After doubling the osmolarity, the NSVs shrunk by 45% in width and by 25% in length (Fig. 2e,f and Extended Data Fig. 3a,b). After halving the osmolarity, there was an increase in NSV width of 45% and in length by 35% (Fig. 2e,f and Extended Data Fig. 3a,b). Under hyperosmotic conditions, the dimensions of connectors did not change, whereas under hypo-osmotic conditions, the connector length increased by 53% but not width. Nevertheless, the experimental observation that NSV width and length correlate inversely with solvent osmolarity is in agreement with the membrane mechanics model, suggesting that membrane pearling instability may underlie the experimentally observed pearled shapes.

Because these osmolarity changes are extreme, the experiments were repeated using more physiologically relevant osmolarity changes (280 to 400 mOsm) by replacing glucose with the membrane-impermeable solute mannitol and varying its concentration (see Methods for details). As expected, the effects were less pronounced, but trends were similar. The hypertonicity causes NSVs to shrink in length by 5% but not in width, whereas decreasing the osmolarity caused the NSVs to expand (increase in width by 7% and increase in length by 11%; Table 1 and Extended Data Fig. 3c–e). Connector dimensions did not change under hypertonic or hypotonic conditions (Table 1 and Extended Data Fig. 3c–e). Therefore, osmotic pressure and thereby membrane tension, in part, regulate pearled axon morphology.

**Extended Data Fig. 4:**
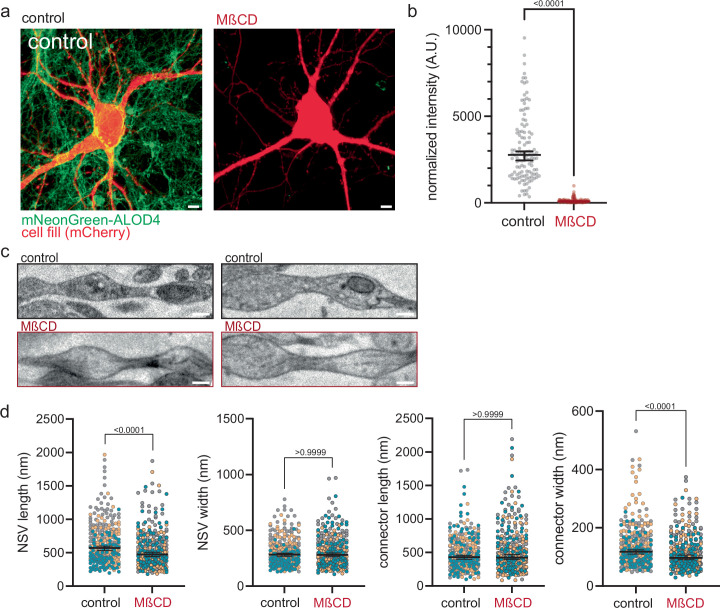
Manipulation of membrane cholesterol causes changes in axonal pearling. **a**. NeonGreen-ALOD4 staining of cultured mouse hippocampal neurons treated with sham (control) or 5 mM MßCD for 30 min. Scale bar = 5 µm. **b**. Plot showing the normalized intensity of NeonGreen-ALOD4. Signals are normalized by the area of each region-of-interest. Control: 2773 ± 234 A.U., MβCD: 86 ± 13 A.U., median and 95% confidence intervals are plotted, Mann-Whitney U test (two-sided), N = 3 independent cultures, n = 127 for control, n = 131 for MβCD. A.U. arbitrary unit. **c**. Additional example micrographs of axons high-pressure frozen after MßCD treatment. Scale bar: 200 nm. **d**. Super Plots of Fig. 2h, showing variability. N = 3 independent cultures and n = 100 axons each. Median and 95% confidence intervals are plotted, Kruskal-Wallis test, followed by Dunn’s multiple comparison test. Each color represents one replicate. Each dot is one axon. Source data

To further test the contribution of membrane mechanics, we manipulated membrane fluidity using methyl-ꞵ-cyclodextrin (MꞵCD; 5 mM for 30 min) to remove cholesterol from the plasma membrane of DIV 21 cultured hippocampal neurons^53,54^. Assuming that cholesterol imparts a general stiffening of the membrane, the removal of cholesterol by MβCD treatment would produce a decrease in membrane bending rigidity, which, in turn, produces a general decrease in NSV size based on modeling (Fig. 2d). Following MꞵCD treatment, the cholesterol level on axons was probed by the exogenously applied domain 4 of anthrolysin O fused with NeonGreen (NeonGreen–ALOD4)^55^. Within 30 min, essentially all accessible cholesterol was removed from the plasma membrane (Extended Data Fig. 4a,b). EM analysis showed that the NSV length decreased under control conditions (12.5% decrease; Table 1, Fig. 2g,h and Extended Data Fig. 4c,d), indicating that membrane fluidity and rigidity also contribute to pearled axon morphology. The remaining dimensions did not change (Table 1, Fig. 2g,h and Extended Data Fig. 4c,d). The concurrence of axon geometry with respect to experimental and model perturbations suggests that membrane mechanics may be a key driver of axon nanopearling.

### Nonmuscle myosin II contributes to pearled axon morphology

**Fig. 3:**
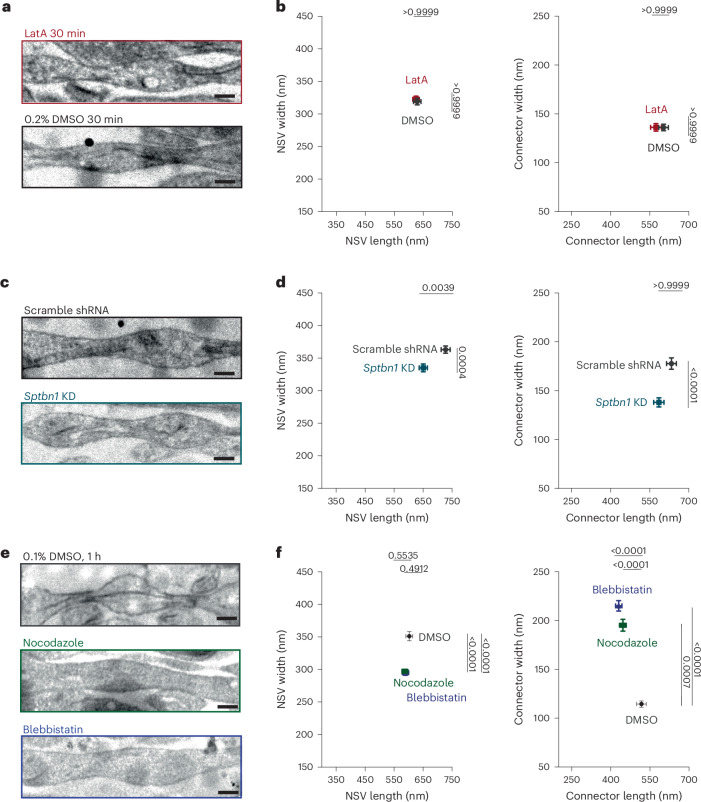
MPS is not sufficient to explain pearled axon morphology. **a**, Example micrographs of axons from cultured mouse hippocampal neurons treated with either 0.2% DMSO or 20 µM LatA for 30 min; scale bars, 200 nm. **b**, Plots showing the dimensions of NSVs (left) and connectors (right) from axons in **a** (DMSO: NSV length 630 ± 13 nm, NSV width 320 ± 5 nm, connector length 600 ± 18 nm, connector width 136 ± 3 nm; LatA: NSV length 230 ± 11 nm, NSV width 320 ± 5 nm, connector length 570 ± 20 nm, connector width 136 ± 4 nm). **c**, Example micrographs of axons from neurons infected with lentivirus carrying either scramble or *Sptbn1* (βII spectrin) shRNA; scale bars, 200 nm. **d**, Plots showing dimensions of NSVs (left) and connectors (right) from axons in **c**. **e**, Example micrographs of axons from neurons treated with 0.1% DMSO, 50 µM nocodazole or 10 µM blebbistatin for 1 h; scale bars, 200 nm. **f**, Plots showing dimensions of NSVs (left) and connectors (right) from axons in **e** (DMSO: NSV length 600 ± 12 nm, NSV width 350 ± 7 nm, connector length 520 ± 19 nm, connector width 115 ± 3 nm; nocodazole: NSV length 590 ± 10 nm, NSV width 300 ± 3 nm, connector length 450 ± 13 nm, connector width 200 ± 6 nm; blebbistatin: NSV length 590 ± 11 nm, NSV width 290 ± 4 nm, connector length 430 ± 12 nm, connector width 215 ± 5 nm). In each experiment, *N* = 3 independent cultures and *n* = 300 axons. Super plots showing variability are available in Extended Data Fig. 5. Data are shown as mean ± s.e.m. All conditions in the figure were analyzed at the same time, and, thus, a Kruskal–Wallis test followed by a Dunn’s multiple comparison test was used. Source data

**Extended Data Fig. 5:**
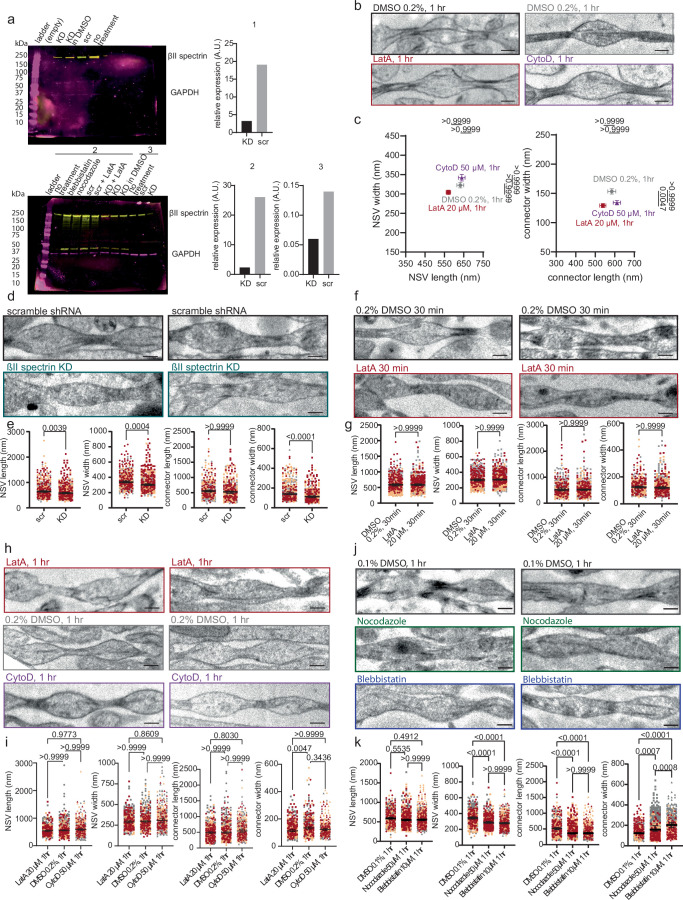
Manipulation of the cytoskeleton causes changes in axonal pearling. **a**. Western blots and quantification showing the efficiency of shRNA-mediated knock down (KD) of ßII spectrin magenta shows ßII spectrin and yellow shows GAPDH. Scr, scramble. **b**. Example micrographs of axons from neurons treated with indicated drugs. Scale bar: 200 nm. **c**. Plots showing dimensions of NSVs (left) and connectors (right) from axons in (a). Mean and SEM are shown. N = 3 independent cultures n = 100 axons each. n.s., not significant. **d**. Additional example micrographs of axons from neurons infected with lentivirus carrying scramble shRNA or shRNA against ßII spectrin. Scale bar: 200 nm. **e**. Super Plots of Fig. 3d, showing experimental variability N = 3 independent cultures and n = 100 axons each. **f**. Additional example micrographs of axons from neurons treated with indicated drugs. Note that these neurons are treated with DMSO or Latrunculin A for 1 hour. Scale bar: 200 nm. **g**. Super Plots, showing experimental variability of Fig. 3a. N = 1 and n = 100 axons. **h**. Additional example micrographs of axons from neurons treated with indicated drugs. Scale bar: 200 nm. **i**. Super Plots of Fig. 3f, showing experimental variability. N = 3 independent cultures and n = 100 axons each. **j**. Additional example micrographs of axons from neurons treated with indicated drugs. Scale bar: 200 nm. **k**. Super Plots, showing experimental variability from experiments in Extended Data Fig. 5j. N = 3 independent cultures and n = 100 axons each. In all super plots median and 95% confidence intervals are shown. Each color represents one replicate. Each dot is one axon. All data analyzed using Kruskal-Wallis test, followed by Dunn’s multiple comparison test. Source data

**Extended Data Fig. 6:**
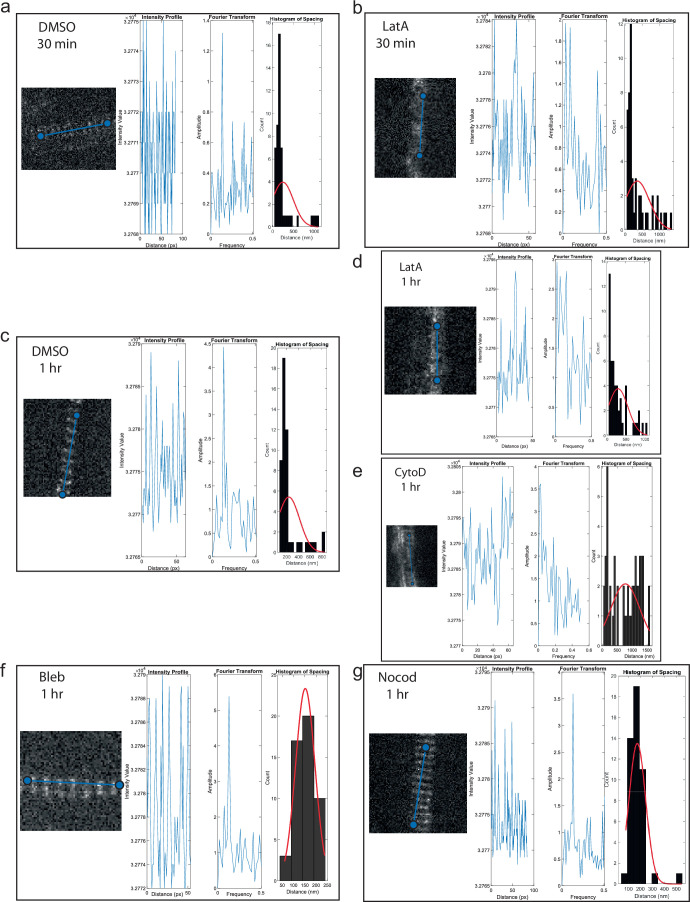
Live STED confirms LatA and CytoD, but not blebbistatin or nocodazole, disrupt actin ring organization. **a–g**. Representative 2D STED images of live axon stained by SiR-actin under control (DMSO) or drug treatments with plotted intensity profile from region indicated by blue line and fast Fourier transform of intensity profile where the largest peak represents the most frequent distance between actin puncta. Histogram (black) and Gaussian distribution (red line) of actin puncta spacing from 50 axons in each condition from 2 independent cultures. **a**, DMSO control, Gaussian distribution values: avg. 245 nm S.D. 253 nm. **b**, LatA 30 min, Gaussian distribution values: avg. 352 nm S.D. 351 nm. **c**, DMSO control, Gaussian distribution values: avg. 235 nm S.D. 184 nm. **d**, LatA 1 hr, Gaussian distribution values: avg. 288 nm S.D. 265 nm. **e**, CytoD 1 hr, Gaussian distribution values: avg. 772 nm S.D. 482 nm. **f**, Blebbistatin 1 hr, Gaussian distribution values: avg. 152 nm S.D. 43 nm. **g**, Nocodazole 1 hr, Gaussian distribution values: avg. 174 nm S.D. 69 nm. Source data

Although the aforementioned model is inspired by membrane mechanics, parameters such as the effective membrane tension may represent other mechanical contributions. Membrane tension is a sum of in-plane tension and cortical cytoskeleton attachment^56–58^. In axons, actin forms periodic rings, termed the MPS, by its interaction with spectrin^24–26^. To discern the contribution of the MPS, we tested the role of different cytoskeletal components (Fig. 3 and Extended Data Fig. 5). We noticed that the frequency distributions did not indicate a close correlation between the 190-nm periodicity of the MPS and the nanopearling (Extended Data Fig. 1g). Nonetheless, to test the contribution of the MPS further, ultrastructural analysis of mouse hippocampal neurons (DIV 21) was performed after treatment with vehicle (0.2% DMSO for 30 min), cytochalasin D (CytoD; 50 µM for 1 h) or latrunculin A (LatA; 20 µM for 30 min or 1 h)^25^, which blocks actin dynamics^59^ and removes the periodic nature of the actin rings^24,25^ (Extended Data Fig. 6a–e) without fully disrupting spectrin^24^. Expression of spectrin β-chain nonerythrocytic 1 (βII spectrin; encoded by *Sptbn1*) was knocked down with short hairpin RNA (shRNA) using lentivirus, as previously described (Extended Data Fig. 5a)^25^. Scramble shRNA was used as a control. These treatments are shown to perturb the MPS^25^. However, axon nanopearling was not altered in neurons treated with LatA or CytoD (Fig. 3a,b and Extended Data Fig. 5b,c,j,k), suggesting that either periodic actin rings do not contribute to nanopearling or the interaction of remaining F-actin with the contractile machinery like myosins can sustain nanopearling in these experiments. Similarly, the pearled morphology remains in *Sptbn1*-knockdown (*Sptbn1*-KD) neurons (Fig. 3c,d and Extended Data Fig. 5d,e), although axons appeared to shrink compared to those treated with scramble shRNA control; NSV length decreased by 10%, whereas the width decreased by 8% (Table 1). The connector width also decreased by 22%, whereas connector length did not change (Table 1). However, the resulting dimensions of axons in *Sptbn1*-KD neurons were similar to those found in wild-type neurons, suggesting that lentiviral infection may have caused enlargement of axons. Nevertheless, the overall axon nanopearling remained after these treatments, suggesting that the static structure of the MPS plays a minimal role in determining axon morphology.

Given that nonmuscle myosin II (NMII) imparts a contractive effect on the axon to potentially induce nanopearling, we next tested the role of NMII by EM. We used the NMII inhibitor blebbistatin (10 µM for 1 h) to block NMII activity without disturbing the MPS^26^ (Extended Data Fig. 6f). After this treatment, nanopearling became less pronounced, particularly at the connectors (NSV width decreased by 23% and connector width increased by 68%; Fig. 3e,f and Extended Data Fig. 5j,k), suggesting that NMII likely restricts the diameter of the connector regions.

Actin–myosin complexes (actomyosin) interplay with microtubules to control cellular architecture^26^. Previous studies show that disruption of microtubules causes excessive axon pearling, also called beading^23,60^, and dissociation of MPS^24^, suggesting a close link between actomyosin and microtubules within axons. Thus, the role of microtubules in axon morphology was tested by inhibiting microtubule polymerization with nocodazole (10 µM for 1 h). At 1 h, the actin rings were not disturbed (Extended Data Fig. 6g). This treatment had little effect on NSVs but decreased the connector length by 20% while increasing the width by 58% (Fig. 3e,f and Extended Data Fig. 5j,k). Together, these results suggest that the cytoskeleton contributes to nanopearling by determining the connector dimensions.

### Axon pearling increases AP dynamic range

**Fig. 4:**
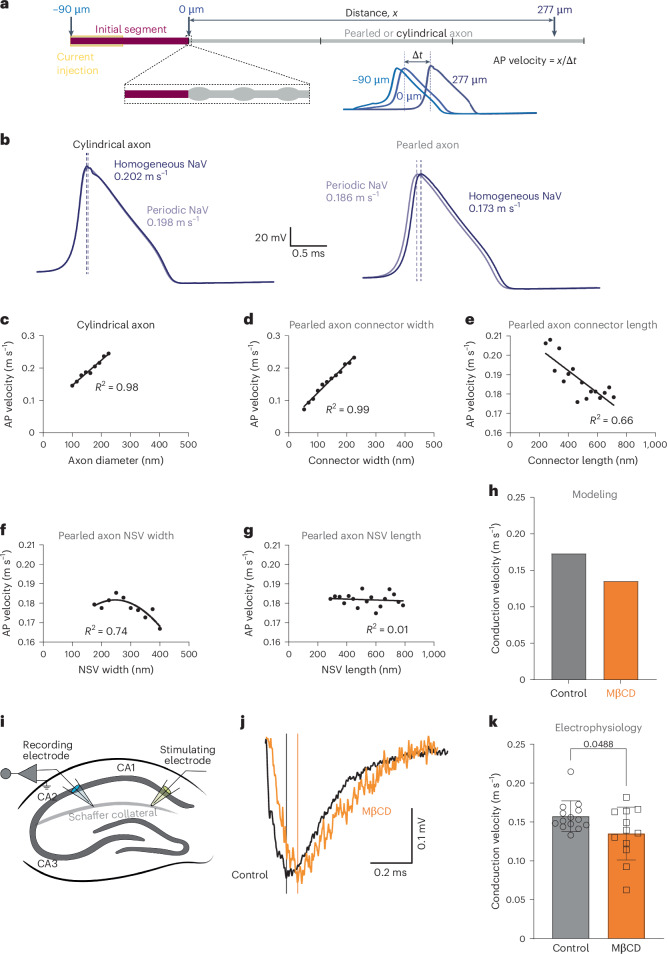
AP propagation relies on axonal morphology. **a**, Schematic showing the model setup. APs were modeled in real geometries using a generalized cable equation to calculate the spatial and temporal distribution of channel current, membrane voltage and gating variables. **b**, Voltage responses at 270 µm from cylindrical axons (left) and pearled axons (right) when NaVs are distributed either uniformly (dark color) or periodically (lighter color). Note that the distribution of NaVs only matters if axons are pearled. **c**, Plot of the relationship between AP velocity and the diameter of cylindrical axons. Dots are fitted with a simple linear regression curve. **d**–**g**, Plots of the relationship between AP velocity and the connector width (**d**), connector length (**e**), NSV width (**f**) and NSV length (**g**). Dots are fitted with a simple linear regression curve, except for **f**, which is fitted by a nonlinear Gaussian curve. **h**, Plot of predicted AP conduction velocity based on the dimensions of NSVs and connectors in neurons treated with sham (control) or 5 mM MβCD. **i**, Schematic of the electrophysiology recording setup. Schaffer collaterals were stimulated from the end of CA1 to measure the back-propagating AP in the CA1. **j**, Example traces from recordings in acute slices of mouse hippocampus treated with either sham (control) or 5 mM MβCD for 30 min. The solid vertical line marks the peak. **k**, Plot of AP conduction velocity from the experiments in **j**. Data were analyzed by Mann–Whitney *U*-test (two sided) and are shown as mean ± s.d.; *N* = 8 animals each, *n* = 14 slices for DMSO, and *n* = 12 slices for MβCD. Source data

To determine how nanopearled axon morphology influences function, we implemented the generalized cable equation with Hodgkin and Huxley currents^1,61^, taking the pearled morphology into account (see Supplementary Methods (Action potential propagation) for details). We simulated electrical conductance over 300 µm of either pearled or cylindrical axon while injecting currents of 30–40 µA cm^–2^ at the first half of an axon initial segment (AIS; Fig. 4a) and measuring the resulting voltage change at the tip of the AIS (−90 µm), at the end of the AIS (0 µm) and near the end of the model axon (277 µm). The conduction velocity was determined based on the time it took for the voltage change to reach its peak at 0 µm and 277 µm (Fig. 4a). Because NaVs are organized by spectrin and ankyrin, which make up the MPS, we also simulated APs with NaVs placed either uniformly along the axon or periodically with an interspacing of 190 nm (ref. ^25^). For cylindrical axons, the periodic or uniform distribution of NaVs did not alter the AP conduction velocity (Fig. 4b), as has been previously demonstrated^62^. However, in nanopearled axons with the average geometries measured in our experiments, the AP was faster if NaVs were periodically distributed by the MPS.

**Extended Data Fig. 7:**
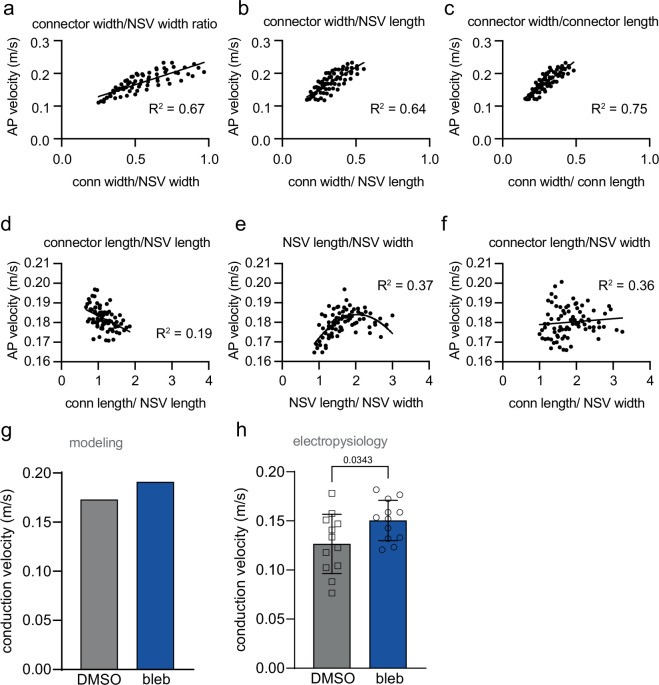
AP velocity varies with axon pearl dimensions. **a-f**, Plots showing the relationship between AP velocity and the ratio of two measured values like connector width/NSV width (**a**), connector width/NSV length (**b**), connector width/connector length (**c**), connector length/NSV length (**d**), NSV length/NSV width (**e**), and connector length/NSV width (**f**). The data are fitted by a simple linear regression in all except for **e**, which is fitted by a gaussian non-linear curve. **g**. Plot showing the predicted AP conduction velocity based on the dimensions of NSVs and connectors in neurons treated with sham (DMSO) or 50 µM Blebbistatin (bleb). **h**. Plot showing the AP conduction velocity from the experiments in (j). Mean and S.E.M. are plotted. Mann-Whitney U test (two-sided). N = 8 animals each, n = 12 slices. Source data

For cylindrical axons, the larger the diameter, the faster the AP conduction velocity (Fig. 4b,c; AP velocity = 0.202 m s^–1^). By contrast, the conduction velocity was highly variable in nanopearled axons depending on the dimensions of the NSVs and connectors. As in cylindrical axons, there was a linear relationship between AP velocity and connector width (Fig. 4d), likely due to the change in axial resistance. Similarly, connector length showed an inverse linear relationship with AP velocity (Fig. 4e). The relationship between NSV width and AP velocity can be described by a concave function, with the AP velocity increasing up to the width of 250 nm, after which it decreases (Fig. 4f). However, no clear correlation was observed between NSV length alone and AP velocity, but the ratio between NSV length and width was a key determinant of AP velocity (Fig. 4g and Extended Data Fig. 7). Thus, based on the local biophysical environment and dimensions of individual NSVs and connectors, the AP velocity can be highly modulated.

To validate our model, we used acute hippocampal slices from P30–P40 mice and recorded fiber volley conduction velocity before and after MꞵCD treatment (5 µM for 30 min). Based on the dimensions of NSVs and connectors in sham- (control) and MꞵCD-treated neurons (Fig. 2g,h), our model predicted APs to be slower in MꞵCD-treated axons (0.135 m s^–1^, ~22% decrease, NSV 573 × 310 nm, connector 567 × 121 nm) than in untreated axons (0.173 m s^–1^, NSV 638 × 309 nm, connector 486 × 136 nm; Table 1 and Fig. 4h). As predicted by the model, the AP velocity decreased from 0.164 ± 0.3 m s^–1^ to 0.117 ± 3 m s^–1^ following MꞵCD treatment (~28% decrease; Fig. 4i–k).

Because cholesterol removal from the membrane by MꞵCD treatment may cause changes to AP conduction unrelated to axon structure, we also tested the effects of blebbistatin treatment (50 µM and 1 h) on fiber volley conduction velocity. Our model predicted APs to be faster in blebbistatin-treated axons as the connectors widen (0.191 m s^–1^ ~10% increase, NSV 590 × 295 nm, connector 431 × 215 nm; Table 1 and Fig. 3). As predicted by the model, the AP velocity increased from 0.127 ± 0.03 m s^–1^ to 0.150 ± 0.02 m s^–1^ following blebbistatin treatment (19% increase; Extended Data Fig. 7g,h). Together, these data suggest a tight correlation between axon nanopearling and function.

### Axon plasticity is induced by biophysical factor modulation

**Fig. 5:**
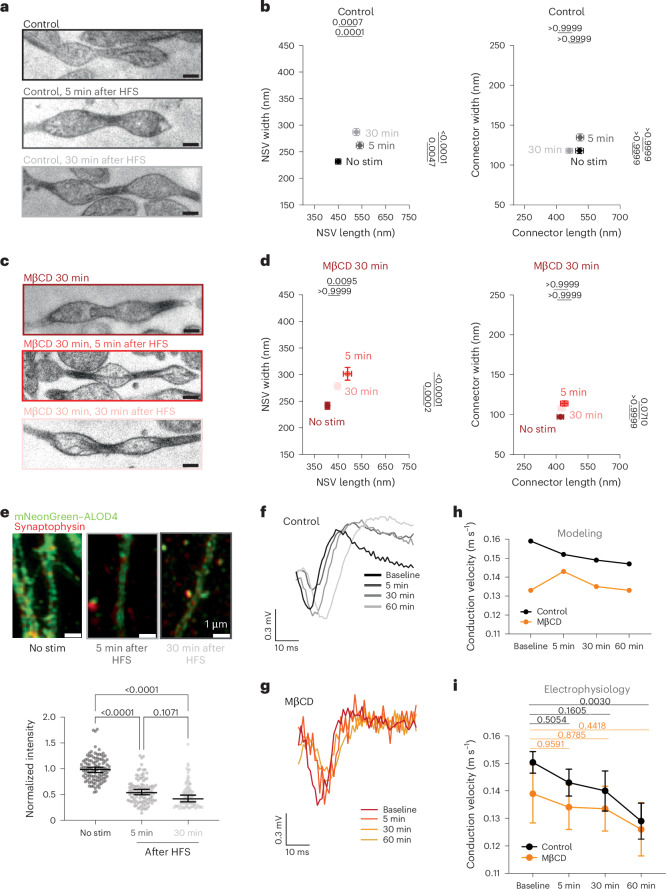
Axonal plasticity is mediated by modulation of membrane mechanics. **a**, Example micrographs showing axon morphology from control neurons unstimulated or stimulated with three trains of 100 pulses at 100 Hz (HFS) and high-pressure frozen at 5 and 30 min after stimulation; scale bars, 200 nm. **b**, Plots showing the dimensions of NSVs (left) and connectors (right) from axons in **a**. Data are shown as mean ± s.e.m. and were analyzed by Kruskal–Wallis test, followed by a Dunn’s multiple comparison test; *N* = 3 independent cultures and *n* = 100 axons each. **c**, Example micrographs showing axon morphology from MꞵCD-treated neurons (5 mM for 30 min) unstimulated or stimulated with three trains of 100 pulses at 100 Hz (HFS) and high-pressure frozen at 5 and 30 min after stimulation; scale bars, 200 nm. **d**, Plots showing the dimensions of NSVs (left) and connectors (right) from axons in **c**. Data are shown as mean ± s.e.m. and were analyzed by Kruskal–Wallis test, followed by a Dunn’s multiple comparison test; *N* = 3 independent cultures and *n* = 100 axons each. **e**, Example images and plots showing normalized intensity of the cholesterol biosensor NeonGreen–ALOD4 in neurons unstimulated or stimulated with HFS and fixed 5 or 30 min after. Data are shown as median and 95% confidence interval (no stimulation (stim): 0.99, 95% confidence interval 0.95–1.04, *n* = 109; 5 min after HFS: 0.54, 95% confidence interval 0.52–0.61, *n* = 110; 30 min after HFS: 0.42, 95% confidence interval 0.44–0.54, *n* = 91); *N* = 3 independent cultures. Data were analyzed by Kruskal–Wallis test, followed by a Dunn’s multiple comparison test. **f**, Example traces from electrophysiology experiments, performed as described in Fig. 4i. **g**, Example traces from electrophysiology experiments in acute slices of mouse hippocampus, performed as described in Fig. 4i. **h**, Predicted AP conduction velocity based on the dimensions of NSVs and connectors before and 5, 30 and 60 min after HFS. **i**, Measured AP conduction velocity before and 5, 30 and 60 min after HFS. Data are shown as mean ± s.e.m. and were analyzed by Kolmogorov–Smirnov test between curves (*P* = 0.23) and Mann–Whitney *U*-test (two sided) between individual time points within treatment conditions. Source data

**Extended Data Fig. 8:**
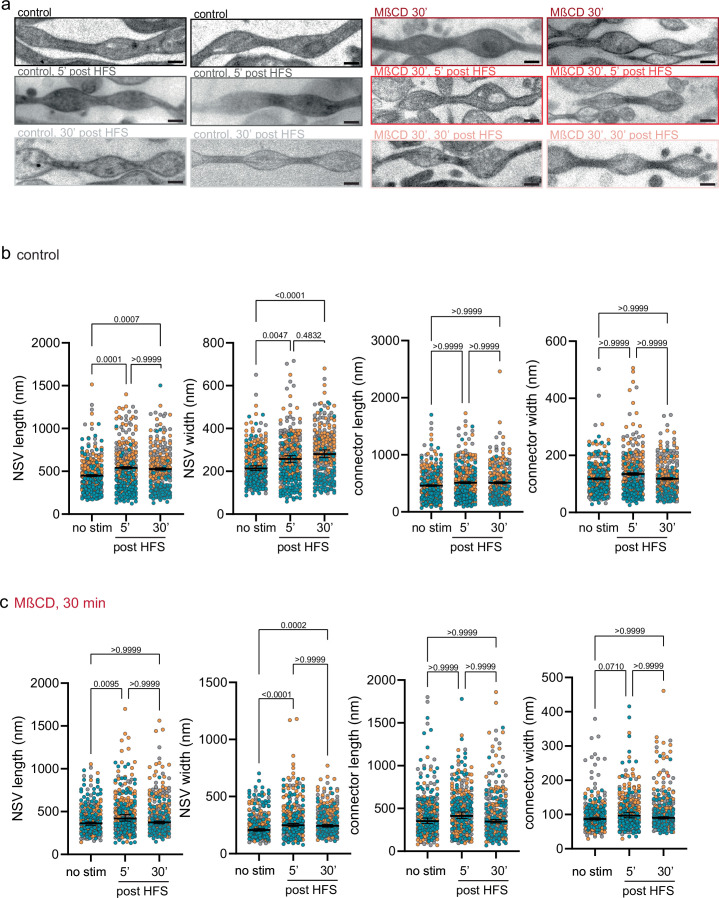
Axonal pearling changes with stimulation even after cholesterol manipulation. **a**. Additional example micrographs showing axon morphology from neurons unstimulated or stimulated with 3 × 100 pulses at 100 Hz (high-frequency stimulation, HFS) and high-pressure frozen at 5 min and 30 min after stimulation. Scale bar: 200 nm. **b**. Super Plots of Fig. 5b, showing experimental variability. N = 3 independent cultures except for control 15’ post stim which was N = 2. n = 100 axons each. **c**. Super Plots of Fig. 5c, showing experimental variability. N = 3 independent cultures. n = 100 axons each. In all super plots median and 95% confidence intervals are shown. N = 3 independent cultures and n = 100 axons each. Each color represents one replicate. Each dot is one axon. All data analyzed using Kruskal-Wallis test, followed by Dunn’s multiple comparison test. Source data

Axon morphology is tightly coupled to neuronal activity and can modulate AP conduction velocity. To better understand how this pearled morphology behaves during sustained neuronal activity, we applied three trains of HFS (100 pulses at 100 Hz, with each train interspaced by 20 s) to DIV 21 dissociated neuronal cultures (Fig. 5 and Extended Data Fig. 8a,b). Ultrastructural analysis showed that NSVs become larger in size 5 min after stimulation (Fig. 5a,b and Extended Data Fig. 8b), showing an 8% increase in length and a 17% increase in width (Table 1). The length and width of connectors did not change (Table 1). These changes are consistent with the previously reported structural plasticity of axons in organotypic hippocampal slice cultures^12^, which persists for a long period following this type of tetanic stimulation. These data suggest that the morphology of axons can be modulated by neuronal activity.

Because the biophysical properties of membranes are key determinants of axon morphology, we next tested whether the membrane properties are altered by monitoring cholesterol levels in the plasma membrane. Indeed, when we imaged cholesterol levels following HFS using NeonGreen–ALOD4 biosensors in wild-type neurons, plasma membrane cholesterol decreased by ~45% immediately after stimulation, and this reduction persisted over 30 min (Fig. 5e). These data suggest that this structural plasticity of axons may arise from changes in levels of cholesterol in the plasma membrane.

If removal of cholesterol from the plasma membrane is solely responsible for the induction, this structural plasticity should be occluded when cholesterol is experimentally removed from the plasma membrane. To test this hypothesis, we treated neurons with 5 mM MβCD for 30 min before stimulation to make membranes more fluid. Interestingly, an increase in NSV dimensions was observed similar to the increase in controls (Fig. 5c,d and Extended Data Fig. 8c), but the NSVs of all MβCD-treated axons were smaller than those from the control and remained smaller than controls even after stimulation (Fig. 5a). These data suggest that modulation of cholesterol levels in the plasma membrane may be linked to structural plasticity, but some other factors are also likely involved in triggering plasticity.

To assess whether axonal structural plasticity induces functional plasticity, we performed AP simulations based on the dimensions obtained in Fig. 5a–d. Our simulations predicted that as axon nanopearling dimensions increase, AP conduction velocity would decrease from 0.159 m s^–1^ to 0.152 m s^–1^ at 5 min, 0.149 m s^–1^ at 30 min and 0.147 m s^–1^ over 60 min in control neurons and remain unchanged over 60 min in MβCD-treated neurons (0.133 m s^–1^ to 0.143 m s^–1^ to 0.135 m s^–1^ to 0.133 m s^–1^, respectively; Fig. 5h). Consistent with the modeling prediction, electrophysiology recordings in acute slices showed that AP conduction velocity decreased following HFS in control samples (baseline: 0.150 ± 0.01 m s^–1^; 5 min: 0.143 ± 0.01 m s^–1^; 30 min: 0.140 ± 0.02 m s^–1^; 60 min: 0.129 ± 0.02 m s^–1^) but did not change in MβCD-treated samples (baseline: 0.140 ± 0.03 m s^–1^; 5 min: 0.134 ± 0.02 m s^–1^; 30 min: 0.133 ± 0.02 m s^–1^; 60 min: 0.126 ± 0.03 m s^–1^; Fig. 5f–i). This change was long lasting, persisting over 60 min, likely due to the expression of long-term potentiation in acute slices. Interestingly, when cholesterol was depleted from acute slices using MβCD, the conduction velocity did not change after HFS (Fig. 5i), suggesting that cholesterol mobilization may indeed be needed for plasticity induction. Together, these results suggest that neuronal stimulation modulates axon nanopearling and function, in part, through the control of biophysical factors.

## Discussion

Extensive study of axon morphology over the last 70 years has revealed increasing complexity in axon structure. Our study uncovers further morphological complexity in that unmyelinated axons in the mammalian central nervous system under near-physiological conditions have a pearls-on-a-string morphology due to membrane-driven instability. Here, we use in silico membrane modeling to show how tension-driven instability governs axon pearling and predict how changes in membrane mechanics would affect pealing behavior. We validated our modeling by showing that treatments that affect membrane mechanics, such as fluctuations in extracellular osmotic pressure, membrane cholesterol concentration manipulation or cytoskeletal manipulation, cause predictable changes in pearling behavior. Importantly, we show that axon pearling greatly impacts AP conduction by both in silico modeling and field potential recordings. We further demonstrate that axon pearl dimensions increase with HFS and that this plasticity changes AP velocity. This structural plasticity is accompanied by a 45% decrease in membrane cholesterol, suggesting a mechanism by which the pearl shape could be altered. Indeed, AP conduction velocity fails to change after artificial cholesterol removal, although some structural plasticity remains. Together, we show that axon morphology is far from simple but instead has complex pearls-on-a-string morphology governed by membrane mechanics that influence AP conduction and plasticity.

Axon pearling is a well-characterized phenomenon that occurs even at the macroscopic level in neurons under stress^23,63–68^. However, the morphology described here is on a nanoscale, with an axon tract ~60 nm in diameter and repeated varicosities ~200 nm in diameter. The difference between the two regions is far below the diffraction limit of light, making ultrastructural characterization essential. However, the use of chemical fixatives for ultrastructural analysis leads to many artifacts in cell and tissue structure^41^. In fact, several studies using cryopreservation techniques have noted a similar morphology in both myelinated and unmyelinated axons^22,69^. Furthermore, intact ctenophore^70^ and *Caenorhabditis elegans*^71^ neurons both exhibit axon nanopearling, indicating that this nanopearling is highly conserved. Thus, we propose that nanopearled morphology is a ubiquitous and prominent feature of axons.

Why do axons pearl? Membrane mechanics studies describe how membrane pearling is caused by the energy minimization of homogeneous membrane tension along cylindrical membrane tubes. Although axons do have complex structure, they are fundamentally membrane tubes, and many of the same biophysical principles are applicable. Consistent with this notion, we found that NSV size is altered by the manipulation of in-plane membrane tension. Additionally, increasing membrane fluidity caused the NSVs to become rounder, showing that membrane properties also control axon nanopearling. However, connectors are less affected by these treatments, suggesting that tension alone may not fully explain axon morphology. Our results here indicate that another component of membrane tension, the cytoskeleton, may be important for regulating nanopearling. Together, these data suggest that axon nanopearling is likely the result of membrane mechanics, particularly in-plane tension, with support from the cytoskeleton.

Recently, the structure of the cytoskeleton in mature axons has been extensively studied to reveal that, unlike other cells, axons have a unique and periodic actomyosin cytoskeleton, the MPS^23–28,30,60,62,72–74^. The motor protein NMII, which binds within one actin ring, serves to dilate the MPS during organelle trafficking and electrical activity^26,74,75^. Many other cytoskeletal-associated proteins, such as NaVs, are anchored to the MPS and are also periodically localized throughout the axon^25^. We report that axon morphology is unaltered when MPS actin is disrupted with LatA or CytoD. We see that axon morphology is also unaltered after *Sptbn1* KD, indicating that static MPS structure is not generating axon nanopearling (Fig. 3a,b and Extended Data Fig. 5b,c,j,k). Considering that LatA treatment affects only a certain pool of F-actin^24,76^, in the absence of actin rings, NMII may still act on the remaining F-actin within the axon (Extended Data Fig. 6b,d) under LatA- or CytoD-treated conditions to maintain morphology. Together, this explains how LatA or CytoD treatment could cause relatively small changes in morphology, whereas inhibiting NMII^26,74^ could more effectively alter axon morphology. Nevertheless, it is important to note that the anchoring of the cytoskeleton to the plasma membrane is not as tight in the axon as in other cell types^29,77^. Thus, cytoskeletal disruption may have a stronger impact in the connector region where the cytoskeleton is in closer contact with the plasma membrane.

Axon nanopearling has strong implications for AP propagation in unmyelinated axons. Previous work with cable theory modeling predicts that sudden changes in axon diameter would slow AP propagation and at a certain size cause AP propagation to fail^4,6,78,79^. In agreement with this theory, our results also suggest that AP conduction velocity is strongly dependent on axon geometry. In particular, connector diameter has a linear relationship with velocity, as the cable theory predicts. However, the relationship between AP velocity and axon morphology is more complicated because of nanopearling. Simultaneous changes between two axon dimensions reveal an optimal NSV length-to-width ratio (~1.7), where AP velocity is at its peak (Extended Data Fig. 7e). Higher and lower than this value would result in slower AP conduction velocity. It is worth noting that the NSVs of neurons from acutely extracted brain tissue are at this ratio, whereas the NSVs of cultured neurons are at about 2. Thus, the surrounding physical environment can influence axon morphology and axon function. Because mechanical properties are likely specific to each neuron type^80^, functional differences across various types of neurons may be attributed, at least in part, to differences in nanopearled axon morphology. Further investigations are warranted.

One very intriguing idea that arises from our study is that direct modulation of biophysical forces and axon morphology could tune AP propagation velocity. AP propagation tuning occurs in myelinated neurons where myelination placement and length are tightly regulated, creating specific firing patterns important for various circuit functions such as coincident detection^81–83^. Through our modeling, we can probe the effect of changing axon nanopearling on AP propagation tuning in unmyelinated axons. In treatments that cause a dramatic change in nanopearling, our modeling predicts a shift in AP propagation velocity. In fact, extracellular osmolarity changes have been seen to change AP firing patterns^84^. Further, Costas et al. has observed an increase in AP velocity from 0.4 m s^–1^ to 0.45 m s^–1^ during NMII inhibition, which causes the actin ring diameter to increase from 300 nm to 400 nm (ref. ^26^). Finally, changes in membrane lipids have also been linked to changes in AP propagation. Recently, Korinek et al. found that cholesterol removal by MβCD treatment disrupts AP propagation by causing AP failure^54^. Similar results were also obtained in crayfish neurons^85^. Our results suggest that cholesterol depletion by MβCD slows down AP propagation velocity. This effect may be due to the direct modulation of axon nanopearling. However, cholesterol also plays an important role in channel clustering, and, thus, axon morphology may not be the sole contributor. Nonetheless, our work suggests a neuronal plasticity paradigm whereby modulation of biophysical factors controls axon nanopearling and thus AP conduction velocity.

## Methods

### Animals

The housing and care of all animals followed the National Institutes of Health animal use guidelines and were approved by the Animal Care and Use Committee at Johns Hopkins University School of Medicine. C57BL/6J mice (wild type; Jackson Laboratory) were used for all experiments. Animals were housed with temperature control at 22 °C on a 12-h light/12-h dark cycle with ad libitum access to food and water.

### Neuron culture

Both males and females were indistinguishably used in this study.

#### Astrocytes

Astrocytes were collected from E18–P0 C57BL/6 cortices with trypsin treatment for 20 min at 37 °C with shaking, followed by dissociation and seeding on T-75 flasks. Astrocytes were grown in full DMEM (DMEM (Gibco, 10569010), 10% fetal bovine serum (Thermo Fisher Scientific, 26140079) and 100 U ml^–1^ penicillin–streptomycin (Gibco, 15140122)) at 37 °C and 5% CO_2_ for 7 days. Two clean 6-mm sapphire disks (Technotrade, 616-100) were placed in each well of a 12-well tissue culture plate (Thermo Fisher Scientific, 720081) and coated with poly-d-lysine (PDL; 1 mg ml^–1^; Sigma, P6407) and collagen (Thermo Fisher Scientific, A1048301). Astrocytes served as a feeder layer for neurons and were seeded at 50,000 astrocytes per well 1 week before hippocampal neuronal culture. FUdR solution (DMEM, 0.81 mM FUdR (Sigma, F0503) and 2.04 mM uridine (Sigma, U3003)) was added to each well and incubated for 2–24 h at 37 °C before seeding neurons.

#### Neurons

E18 C57BL/6 embryos were decapitated and stored in HBSS (Gibco, 14175095). The hippocampi from each brain were removed in dissection medium (1× HBSS, 100 U ml^–1^ penicillin–streptomycin, 1 mM pyruvate (Gibco, 11360070), 10 mM HEPES (Gibco, 15630080) and 30 mM glucose (Sigma, G6152-100G)). Two hippocampi were then placed in the same 15-ml tube with dissection medium, and 0.01% DNaseI (Sigma, DN25) and 10 U ml^–1^ papain (Worthington, LS003119) were added and incubated at 37 °C with gentle perturbation every 5 min for 20 min. The tissue was dissociated by gentle titration and run through a 70-μm cell strainer (Thermo Fisher Scientific, 22363548). Cells were spun down at 120*g* for 2 min and gently resuspended in NM5 (Neurobasal medium (Gibco, 21103049), 5% horse serum (Gibco, 26050088), 100 U ml^–1^ penicillin–streptomycin, 2% GlutaMAX supplement (Gibco, 35050061) and 2% B27 (Gibco, 17504044)). Cells were seeded on sapphire discs in 12-well plates at 75,000 cells per well or in 6-well plates at 200,000 cells per well in NM5.

The next day, the medium was changed to NM0 culture medium (Neurobasal medium, 2% GlutaMAX supplement and 1% B27). A half-medium change was done at DIV 14 with fresh NM0.

#### Organotypic hippocampal slice culture for high-pressure freezing

Organotypic hippocampal slice cultures were performed using a protocol modified from Qian et al.^86^. C57BL/6 mouse pups were killed at P5–P8 by rapid decapitation. Brains were collected and dissected in ice-cold dissection medium (MEM, 24 mM HEPES and 10 mM Tris-Cl). Forebrains were isolated and cut into 300- to 400-μm-thick coronal slices using a McIlwain tissue chopper (Ted Pella). Hippocampal slices were gently detached from the forebrain slices using forceps and placed onto Millicell inserts (30 mm in diameter, 0.4-μm pore size). Three to four slices were placed on a single insert. After carefully removing excess liquid around the tissue slices, the inserts were placed into six-well plates containing slice culture medium (50% MEM, 25% heat-inactivated horse serum and 25% HBSS), with the membrane of the inserts just touching the surface of the medium. The slices were maintained in a 37 °C humidified incubator with 5% CO_2_. Medium in the plate was replaced with a low-serum medium (5% horse serum) at DIV 1 and changed every 2 days from that point on.

#### Organotypic slice cultures for live STED microscopy

Organotypic hippocampal slices (Gähwiler type) from C57BL/6 P5–P7 wild-type mice were dissected and cultured for 3–5 weeks in a roller drum at 35 °C (ref. ^87^). Experimental procedures were in accordance with the European Union and CNRS UMR 5297 institutional guidelines for the care and use of laboratory animals (Council Directive 2010/63/EU) and were approved by the Committee of Ethics of Bordeaux (50120198-A).

#### Acute cortical tissue extraction

Tissue from the neocortex of adult mice was prepared in the lab of G.K., with ethical approval from the Swiss Federal Veterinary Office (experimentation license 1889.3). Adult mice (C57BL/6, 8 weeks old) were decapitated, and, using scissors and forceps, the brain was immediately exposed. A piece of cortex was removed using forceps and placed on top of a closed plastic Petri dish containing ice. The tissue was then sliced with razor blades to produce 200-µm-thick slices.

#### Chemical fixation

Adult mice (C57BL/6, 8 weeks old) were deeply anesthetized with inhalation anesthetic (isoflurane) and immediately perfused with a buffered solution of 2.5% glutaraldehyde and 2% paraformaldehyde in phosphate buffer (0.1 M (pH 7.4), 250–300 ml per animal). One hour after perfusion, the brain was removed, and 80-µm-thick slices were cut using a vibratome. The slices were washed in cacodylate buffer (0.1 M (pH 7.4), three times for 5 min) and postfixed in 1% osmium tetroxide and 1.5% potassium ferrocyanide in cacodylate buffer (0.1 M (pH 7.4), 40 min). Samples were then stained with 1% osmium tetroxide in cacodylate buffer (0.1 M, pH 7.4) for 40 min and then in 1% uranyl acetate for 40 min before being dehydrated in a graded alcohol series (3 min each change) and embedded in Durcupan resin. Specimens were cured at 60 °C for 24 h.

### Drug treatments

#### LatA

LatA (Tocris Bioscience, 3973100U) was dissolved in DMSO to a stock concentration of 10 mM, added to the cells to a final concentration of 20 µM and incubated in a cell incubator for the indicated times before the experiment. Stocks were used within 1 week.

#### CytoD

CytoD (Tocris Bioscience, 1233) was dissolved in DMSO to a stock concentration of 25 mM, added to the cells to a final concentration of 50 µM and incubated in a cell incubator for 1 h before the experiment. Stocks were used within 1 month.

#### Blebbistatin

(±)-Blebbistatin (Abcam, ab120425) was dissolved in DMSO to a stock concentration of 10 mM, added to the cells to a final concentration of 10 µM and incubated for 1 h in a cell incubator before the experiment. Stocks were used within 1 week.

#### Nocodazole

Nocodazole (Tocris Bioscience, 122810) was dissolved in DMSO to a stock concentration of 50 mM, added to the cells to a final concentration of 50 µM and incubated for 1 h in a cell incubator before the experiment. Stocks were used within 1 week.

#### MβCD

MβCD was dissolved directly in the medium to a final concentration of 5 mM and incubated for 30 min in a cell incubator before the experiment.

### Lentivirus production and infection

HEK293T cells (1.2 × 10^6^ cells per flask; ATCC, CRL-3216) were plated in T-75 flasks (Sarstedt, 100437) coated with PDL (10 µg ml^–1^) in full DMEM and incubated in 37 °C for 3 days. Cells were then trypsinized with 0.05% Trypsin-EDTA (Sigma, T1426) and replated at 6.5 × 10^6^ cells in 10 ml of DMEM in a new PDL-coated T-75 flask. When cells were ~90% confluent, the medium was switched to NBA medium (1% GlutaMAX, 2% B27 and 0.2% penicillin–streptomycin). The modified shuttle vector (FUGW)^88^ containing expression constructs and helper plasmids (VSV-G and CMV-dR8.9) was mixed at 20, 5 and 7.5 µg, respectively, in 640 µl of NaCl solution (150 mM; solution I). Another solution (solution II) was prepared as follows: 246.7 µl of water, 320 µl of NaCl (300 mM) and 73.3 µl of polyethylenimine (0.6 µg µl^–1^; Polysciences, 24765-2). Solutions I and II were combined and incubated at 24 °C for 10 min, followed by addition to the T-75 flask containing HEK293T cells. Cells were incubated at 37 °C (5% CO_2_) for 3 days. The medium containing lentivirus was collected, and virus particles were concentrated 20-fold using Amicon Ultracel-100,000 centrifugal filter units (EMD Millipore, 901024). For all KD experiments, dissociated hippocampal neurons were infected on DIV 9 with lentiviruses carrying the expression constructs. For all live-imaging experiments, dissociated hippocampal neurons were infected on DIV 5 with lentiviruses carrying the expression constructs.

### shRNA constructs

To express our shRNA in neurons, lentiviral expression constructs were used. All vectors were based on the lentiviral shuttle vector FUGW^88^. The two sense sequences of *Sptbn1* shRNA were 5′-GCATGTCACGATGTTACAA-3′ and 5′-GGATGAAATGAAGGTGCTA-3′, as previously described^24^, and were inserted using the Infusion HD Cloning kit (Takara Bio, NC1470242).

### Viral infection for STED

For specifically labeling CA3 neurons, a glass micropipette backfilled with Sindbis–GFP viral particles diluted in TNE buffer (0.1 M NaCl, 0.05 M Tris-Cl (pH 8), 0.5 M EDTA and 0.001% Tween-20) connected to a pressure-injection device (Picospritzer, Parker) was used. The pipette was positioned into the CA3 pyramidal layer, and the virus was injected by brief pressure pulses (50–150 ms; 10–15 psi), yielding an infection of approximately 30–50 neurons. Experiments were conducted 36–48 h after the infection, giving sufficient GFP expression while preserving the physiological health of the cells.

### Western blotting

Western blotting was used to verify KD efficacy. Protein lysates were obtained from cultures of hippocampal neurons infected with the shRNA-containing lentiviral construct. Briefly, cells were lysed using RIPA lysis buffer (Pierce, 89900). Proteins were separated by SDS–PAGE (Bio-Rad, 4561095) and transferred to nitrocellulose membranes (Bio-Rad, 1620115). Membranes were blocked with 5% milk and incubated with either rabbit anti-β-actin (1:5,000; SYSY, 251003) or rabbit anti-GAPDH (1:1,000; Abcam, ab37168) as a loading control and mouse anti-βII spectrin (1:1,000; BD Cell Analysis, BDB612563) overnight at 4 °C. Secondary antibody was added (Li-COR IRDye 800 cw, goat anti-mouse, 925-32210, 1:30,000; Li-COR IRDye 680RD, goat anti-rabbit IgG (H + L) 925-68071, 1:30,000; 1 h at room temperature), and results were imaged using a Li-COR ODYSSEY CLx (0958) and analyzed using Image Studio version 5.2 software.

### Cholesterol sensor cloning and purification

NeonGreen–ALOD4 was used to stain plasma membrane cholesterol in cultured hippocampal neurons. ALOD4 (Addgene, 111026) was N-terminally tagged with NeonGreen. Codon-optimized NeonGreen was synthesized as gblock DNA from Integrated DNA Technologies. NeonGreen–ALOD4–His_6_ was cloned in pET28a vector backbone for bacterial expression. The plasmid was transformed into *Escherichia coli* BL21 DE3 Rosetta cells, and protein expression was induced with 0.5 mM IPTG for 16 h at 4 °C. Histidine-tagged NeonGreen–ALOD4 was purified using an AKTA purifier (GE). Briefly, cells were lysed using B-PER (Thermo Fischer Scientific, 78243) cell lysis buffer supplemented with benzonase (25 U ml^–1^), MgCl_2_ (2 mM), ATP (2 mM), imidazole (20 mM) and protease inhibitor (cOmplete, EDTA free, Roche). Cell debris was removed by centrifuging at 112,000*g* for 20 min at 4 °C. The clear lysate was loaded onto 1-ml HisTrap FF (Cytiva) columns pre-equilibrated with buffer A (20 mM HEPES (pH 7.4), 150 mM NaCl and 20 mM imidazole). After washing the column with 10 volumes of buffer A, protein was eluted with a gradient of 20–500 mM imidazole. The purity of the protein was confirmed by SDS–PAGE stained with Coomassie blue. More than 90% purity was achieved, and protein was stored in 20% glycerol as a 40 µM stock solution.

### Cholesterol staining of neurons

To visualize cholesterol, hippocampal neurons (DIV 21) were stained with NeonGreen–ALOD4 (1 µM final concentration) for 30 min at 37 °C. For depleting cholesterol from the neuronal plasma membrane, cells were treated with 5 mM MβCD (freshly dissolved as 6 mg of powder per 1 ml of NM0 medium) and incubated for 30 min at 37 °C. MβCD-containing medium was removed by a quick wash with fresh medium, and 1 µM NeonGreen–ALOD4 was added to the cells and allowed to bind for 30 min at 37 °C. After the binding reaction, cells were briefly washed with medium, fixed with 4% sucrose and 4% paraformaldehyde in PBS and used for further antibody labeling.

For HFS experiments and cholesterol labeling, neurons were cultured on grid coverslips (iBiDi, Grid 50). On DIV 21, coverslips were placed in an RC-BRFS slotted perfusion chamber with field stimulation (Warner Instruments), and cells were perfused with physiological saline solution (140 mM NaCl, 2.4 mM KCl, 10 mM HEPES, 10 mM glucose (pH adjusted to 7.3 with NaOH), 4 mM CaCl_2_ and 1 mM MgCl_2_, 300 mOsm). The cells were stimulated with three trains of 100 pulses at 100 Hz, with each train interspaced by 20 s, and either recovered for 30 min in a CO_2_ incubator at 37 °C or directly used for cholesterol labeling. For the no stimulation control (sham), cells were placed in the perfusion chamber for the same amounts of time and recovered in medium for 30 min. All treated cells were labeled with 1 µM NeonGreen–ALOD4 for 30 min.

### Immunofluorescence and Airyscan imaging

For Airyscan imaging, samples were imaged on a Zeiss LSM880 (Carl Zeiss) in Airyscan mode. Fluorescence was acquired using a ×63/1.4-NA objective lens at a pixel size of 2,048 × 2,048 with the following settings: pixel dwell time of 1.02 ms and pinhole size above the lower limit for Airyscan imaging, as computed by ZEN software.

For experiments examining axon morphology, synapses were marked with RIM1a–eCFP overexpression construct packaged in a lentivirus. The cytosol was labeled using a cell-fill HaloTag in a lentivirus construct. JFX554 Halo-ligand dye (final concentration of 100 nM) was added to the culture medium 30 min before imaging. Directly before imaging, cells were washed with fresh hibernate E with 2% GlutaMAX and imaged at 37 °C. After a region with individual axons visible was selected, one image was acquired before removing the medium and replacing it with 37 °C 4% paraformaldehyde + 4% sucrose. Fixed images were acquired 5 min after fixative was added.

For experiments comparing fluorescence intensities between no treatment and MβCD-treated cells, staining and microscope settings remained constant. NeonGreen–ALOD4 was used to stain cholesterol, and NeonGreen signal intensity was used to determine cholesterol level. Presynaptic regions were determined with either synaptophysin or bassoon. Axons were distinguished from dendritic processes based on their morphology, thin and no spines. *Z* sections (0.18 µm) were acquired for each presynaptic bouton along axons. NeonGreen intensities, depicting plasma membrane cholesterol levels in varicosities, were quantitated in ImageJ.

For measuring cholesterol levels in cells treated with or without MβCD, synaptophysin (SYSY, 101011; 1:100) was used to identify synapses, and NeonGreen intensity was measured in ImageJ. Signal was normalized to the area of the selected axons. For HFS experiments, the axonal marker ankyrin G (SYSY, 386004; 1:100) was used to determine the regions of interest. All primary antibody incubations were performed overnight at 4 °C, and all secondary antibodies were used at 1:500 for 45 min. For HFS experiments, fluorescence intensity of the cholesterol sensor was normalized to ankyrin G signal and was expressed as the percentage of no treatment control.

### STED microscopy

Note that the cell-fill STED data (Fig. 1e and Extended Data Fig. 1g) used here were collected in a previous study^12^ and were reanalyzed considering our findings on axon nanopearling. For the detailed imaging methods, please refer to Chéreau et al.^12^. Briefly, we used a home-built STED microscope based on an inverted microscope (DMI 6000 CS Trino, Leica) using a galvanometric beam scanner (Yanus IV, TILL Photonics) and a high-numerical-aperture objective lens (Plan Apo, ×100/1.4-NA oil, Leica). The software Imspector (A. Schönle, MPI for Biophysical Chemistry) controlled image acquisition, and parameters that minimize photodamage and photobleaching were chosen. All images were acquired with time-averaged powers of <6 μW for excitation and <8 mW for STED (measured at the back aperture of the objective). Image stacks were acquired with a voxel size of 19.5 nm (*x*, *y*) and 375 nm (*z*) and a dwell time of 15 μs. A piezo *Z*-focusing device (Physikinstrumente) controlled imaging depth, which was maximally 15 μm below the tissue surface. *Z* stacks of 40 × 40 × 3 μm (*x*, *y* and *z*) were acquired every 6 min.

For imaging actin in the axon, STED images were obtained using an Abberior Facility. The STED beam (775 nm) was set at 40% power with a pixel size of 25 nm, a dwell time of 5 µs and a pinhole size of 1 AU, as calculated by the Imspector software (Abberior Instruments Development Team, Imspector Image Acquisition & Analysis Software v16.3, http://www.imspector.de). A 60×/1.42-NA oil objective was used, and the excitation wavelength (640 nm) was imaging 25% power. The fluorescent photons were detected by two avalanche photodiodes (SPCM-AQR-14-FC, PerkinElmer), and images were obtained by scanning a piezo-controlled stage (Olympus, IX83) controlled with the Imspector data acquisition program. To label actin, SiR–actin (cytoskeleton, CY-SC001) was dissolved in DMSO to a stock concentration of 1 mM and added to the imaging medium (hibernate E + 2% GlutaMAX) to a final concentration of 2 µM, which replaced the medium on the cells 10 min before imaging. Imaging was performed at room temperature and lasted no longer than 30 min. Images were analyzed in MATLAB using a custom script available at https://github.com/shigekiwatanabe/axon_pearl_manuscript.

### High-pressure freezing and freeze substitution for dissociated cell culture and organotypic slice cultures

#### Dissociated cell culture

Cells cultured on sapphire disks were frozen using a high-pressure freezer (EM ICE, Leica Microsystems). Each disk with neurons was transferred into physiological saline solution (140 mM NaCl, 2.4 mM KCl, 10 mM HEPES, 10 mM glucose (pH adjusted to 7.3 with NaOH), 4 mM CaCl_2_ and 1 mM MgCl_2_; 300 mOsm) except when under hyperosmotic and hypo-osmotic conditions, when the saline solutions contained the following: 280 mM NaCl, 4.8 mM KCl, 20 mM HEPES, 20 mM glucose, 4 mM CaCl_2_ and 1 mM MgCl_2_ (600 mOsm); 140 mM NaCl, 2.4 mM KCl, 10 mM HEPES, 2 mM glucose, 4 mM CaCl_2_, 1 mM MgCl_2_ and 108 mM mannitol (400 mOsm); 140 mM NaCl, 2.4 mM KCl, 10 mM HEPES, 2 mM glucose, 4 mM CaCl_2_, 1 mM MgCl_2_ and 15 mM mannitol (300 mOsm); 140 mM NaCl, 2.4 mM KCl, 10 mM HEPES, 2 mM glucose, 4 mM CaCl_2_ and 1 mM MgCl_2_ (280 mOsm) and 70 mM NaCl, 1.2 mM KCl, 5 mM HEPES, 5 mM glucose, 4 mM CaCl_2_ and 1 mM MgCl_2_ (150 mOsm). Assembly was performed in the freezing chamber maintained at 37 °C. The polycarbonate sample cartridges (Leica, 16771881, 16771882 and 16771838) were also warmed to 37 °C. Immediately after, the sapphire disk was mounted on the sample holder, and the cartridge was inserted into the freezing chamber. The disk was mounted onto the middle plate with neurons facing down into a specimen carrier, 100-µm side up (Technotrade, 610-100). A 200-µm spacer ring (Technotrade, 1259-100) was placed on top of the sapphire disk. The entire assembled middle plate was then placed on a piece of filter paper to remove the excess liquid, loaded between two half cylinders and transferred into the freezing chamber. The frozen sample was automatically dropped into a storage Dewar filled with liquid nitrogen. After freezing, the middle plate with sapphire disks was transferred to a cup containing anhydrous acetone (−90 °C), which was placed in an automated freeze substitution system (EM AFS2, Leica Microsystems) using prechilled tweezers. Samples were transferred to specimen holders (Leica, 1670715 and 16707154) containing fixative (1% glutaraldehyde (Electron Microscopy Sciences, 16530) and 0.1% tannic acid (Sigma, 403040-100G) in acetone) and precooled to −90 °C in the AFS. The following freeze substitution program was used: incubate at −90 °C for 44 h, five washes in prechilled acetone (30 min per wash), switch to 2% osmium tetroxide (Electron Microscopy Sciences, 19132) in acetone and incubate at −90 °C for an additional 41 h, −90 to −20 °C in 14 h, −20 °C for 12 h and −20 to 4 °C in 2 h.

#### Organotypic slice culture

For high-pressure freezing experiments, the areas on the Millicell insert membrane where the hippocampal slices were placed were cut out with a scalpel. The slice along with the membrane at the bottom was soaked in cryoprotectant (140 mM NaCl, 2.4 mM KCl, 10 mM HEPES, 10 mM glucose, 2 mM CaCl_2_, 3 mM MgCl_2_ and 20% bovine serum albumin) and placed onto a 6-mm sapphire disk. High-pressure freezing was performed as described above using a Leica EM ICE device except freeze substitution was performed in a fixative solution containing 1% osmium tetroxide, 1% methanol, 0.1% uranyl acetate and 3% water dissolved in acetone. Samples were held at −90 °C for 24 h, raised to −25 °C at a rate of 5 °C h^–1^, held at −25 °C for 12 h, raised to 0 °C at a rate of 5 °C h^–1^, held at 0 °C for 1 h and finally raised to 20 °C at a rate of 20 °C h^–1^. Samples were then washed with acetone, stained en bloc with 1% uranyl acetate and washed again with acetone.

#### Embedding and sectioning for dissociated cell culture and organotypic slice cultures

Following freeze substitution, samples were washed with anhydrous acetone four times for 10 min each at room temperature. After washing, samples were infiltrated with 30% epon araldite, followed by 70% epon araldite (6.2 g epon, 4.4 g araldite, 12.2 g DDSA and 0.8 ml of BDMA; Ted Pella, 18012) in anhydrous acetone every 2 h. The samples were then transferred to caps of polyethylene BEEM capsules (EMS, 102096-558) with 90% epon araldite and incubated overnight at 4 °C. The next day, samples were moved into new caps with fresh 100% epon araldite every 1 h three times, after which resins were cured at 60 °C for 48 h. Once cured, sapphire disks were removed from the resin, leaving the cells behind. The block was then cut and fixed onto a dummy block using super glue for sectioning. Forty-nanometer sections were cut using an ultramicrotome (EM UC7, Leica microsystems) and collected on single-slot copper grids (Ted Pella, 1GC12H) coated with 0.7% pioloform in chloroform for transmission EM imaging or on silicon wafers. The sections were stained with 2.5% uranyl acetate (Ted Pella, 19481) in 75% methanol.

#### High-pressure freezing, freeze substitution, embedding and sectioning for acutely extracted brain bits

Acutely extracted brain bits were placed inside aluminum sample holders (6 mm in diameter) with a 200-µm-deep cavity. A drop of 1-hexadecene was added to ensure that no air was trapped once the sample holder was closed. This was high-pressure frozen using a Leica EM HPM100 (Leica Microsystems). The procedure was completed in less than 90 s from the moment of decapitation. The frozen samples were then stored in liquid nitrogen until further processing.

Frozen samples were embedded at low temperature (AFS2, Leica Microsystems). They were first exposed to 0.1% tannic acid in acetone for 24 h at −90 °C, followed by 12 h in 2% osmium tetroxide in acetone at the same temperature. The temperature was then raised to −30 °C over 72 h, the liquid was replaced with pure acetone, and the temperature was increased to −10 °C over 24 h. The tissue samples were embedded in resin by first mixing with a 30% resin in acetone mix from −10 to 0 °C, a 50% mix from 0 to 10 °C, a 70% mix from 10 to 20 °C and then 100% resin at 20 °C (2 h for each step for a total of 8 h). Fresh resin was added for a further 2 h and then placed in silicon molds for 24 h at 65 °C for the resin to harden.

### Transmission EM

For cultured neurons and organotypic slice cultures, samples were sliced into 40-nm sections on a Leica Ultramicrotome 7 and collected on pioloform-coated single-slot grids for transmission EM. For organotypic slice cultures, serial sections were also collected on silicon wafers for array tomography imaging. These sections were imaged at 80 kV at a magnification of ×20,000 on a Hitachi 7600 transmission electron microscope equipped with a dual AMT CCD camera system. Images were acquired through AMT Capture v6. Each sample was given a random number, and about 50 electron micrographs per sample were acquired.

For both high-pressure frozen and chemically fixed acute tissue in resin, serial sections (50–100 sections per ribbon) from the tissue samples were collected on single-slot copper grids with a pioloform support film. Images were collected in series using a transmission electron microscope (Tecnai Spirit, FEI Company) operating at 80 kV and housing a digital camera (FEI Eagle, 4k × 4k).

### Array tomography

The serial sections were imaged using a scanning electron microscope, specifically a JSM-7900F (JEOL) with a backscattered electron detector. Sections were imaged automatically with Array Tomography Supporter, a custom software for serial-section scanning electron microscopy, also called array tomography^89^, at an acceleration voltage of 7 kV with a resolution of 2.0 nm per pixel.

### EM image analysis

Each set of images from a single experiment were shuffled for analysis as a single pool using a custom R (R Development Team, R Studio 1.3, R version 3.5.1) script. Images that did not contain an axon or could not be reliably segmented were excluded from segmentation after images were randomized. No other data were excluded. Structures were considered axons if they either contained a presynaptic terminal or were smaller than 1 µm in diameter. Varicosities with a cluster of synaptic vesicles or those that were isolated from axonal processes were not measured in this study. NSVs and connectors were measured in Fiji (version 1.54e) using a custom macro. Any varicosities containing 40-nm-diameter vesicles were excluded as synaptic varicosities. To minimize any possible bias and to maintain consistency, all image segmentation, still in the form of randomized files, was checked by a second lab member. Dimensions were then quantitated using custom MATLAB (MathWorks, R2017-R2021a) scripts (available from https://github.com/shigekiwatanabe/axon_pearl_manuscript). For three-dimensional data, dimensions were determined as follows. First, dimensions in two dimensions were calculated as described above, and then three dimensions were calculated using the Pythagorean theorem with the assumption that each image was aligned and each section was of equal thickness. All example micrographs shown had their brightness and contrast adjusted to be similar depending on the raw image, rotated and cropped in Adobe Illustrator.

### Electrophysiology

Adult mice of both sexes ranging from 6 to 8 weeks of age were anesthetized using a combination of isoflurane inhalation and avertin injection. Mice underwent cardiac perfusion using chilled sucrose solution (10 mM NaCl, 2.5 mM KCl,10 mM glucose, 84 mM NaHCO_3_, 120 mM NaH_2_PO_4_, 195 mM sucrose, 1 mM CaCl_2_ and 2 mM MgCl_2_) saturated with 5% oxygen/95% carbon dioxide (carbogen). Brains were rapidly dissected, and hippocampi were removed. Hippocampi were then embedded in premade agarose molds and sliced at 400 µm using a Leica VT1200S vibratome at a speed of 0.05 mm s^–1^ and amplitude of 1.0 mm. Slices were then transferred to artificial cerebrospinal fluid (ACSF; 119 mM NaCl, 2.5 mM KCl, 1.3 mM MgSO_4_, 2.5 mM CaCl_2_, 26 mM NaHCO_3_, 1 mM NaH_2_PO_4_ and 11 mM d-glucose; 315 osM, pH 7.4) heated using a water bath to 32 °C and saturated with carbogen. Slices were recovered at this temperature for 15 min before removal from the bath and recovery for 1 h at room temperature.

Recordings were performed at 32 °C in ACSF containing 2 mM kynurenic acid to suppress excitatory postsynaptic potentials. Slices treated with MβCD (5 mM in ACSF) were preincubated 30 min before recording. MβCD was also present in recording ACSF. Slices treated with blebbistatin (50 µM in ACSF from a 10 mM stock in DMSO) were preincubated 1 h before recording. Blebbistatin was also present in recording ACSF. For the controls in the blebbistatin treatment, slices were preincubated with 0.1% DMSO 1 h before recording. DMSO was also present in the recording ACSF. Glass pipettes containing silver chloride electrodes were used to both stimulate and record. The stimulating electrode was filled with ACSF and placed in CA1 to stimulate the Schaffer collaterals. The recording electrode was filled with 1 M NaCl and placed at varying distances ranging from 0.2 to 1 mm away from the stimulating electrode toward CA2/CA3 in CA1 Schaffer collaterals. A bipolar square pulse of 0.3 ms at 60 mV was applied every 1 min for 20 min and averaged to determine the baseline. Long-term potentiation was induced by applying three trains of 100 pulses at 100 Hz separated by a 20-s interval. A bipolar square pulse of 0.3 ms at 60 mV was applied every 1 min for 1 h after long-term potentiation induction. Recordings were taken using Multiclamp 700B and Digidata 1550B and Clampex v11.2 software. The stimulus was applied using A-M Systems Isolated Pulse Stimulator Model 2100. One to three slices per condition per mouse were recorded. One collection of data was excluded from analysis in an acute slice where many of the neurons in the slice were going though apoptosis during the final recording. Traces were analyzed using Clampfit software v11.2, and fiber volley speed was determined (time to peak/distance traveled) using a custom MATLAB script.

### Statistical analysis

All distributions shown of NSVs and connectors were pooled from multiple experiments. All data were initially examined on a per experiment basis (within a freezing performed on the same day and all segmentation performed in a single randomized batch). We did not predetermine sample sizes using power analysis but based them (*N* = 2 and 3, *n* > 100) on our prior studies^90–94^. All data were tested for normality by the D’Agostino–Pearson omnibus test to determine whether to use parametric or nonparametric methods. Comparisons between multiple groups followed by full pairwise comparisons were performed using one-way analysis of variance followed by Kruskal–Wallis test followed by Dunn’s multiple comparisons test. All statistical analyses were performed and all graphs were created in GraphPad Prism.

### Morphology simulations

The procedures are described in the Supplementary Information.

### AP modeling

The procedures are described in the Supplementary Information.

### Reporting summary

Further information on research design is available in the Nature Portfolio Reporting Summary linked to this article.

## Online content

Any methods, additional references, Nature Portfolio reporting summaries, source data, extended data, supplementary information, acknowledgements, peer review information; details of author contributions and competing interests; and statements of data and code availability are available at 10.1038/s41593-024-01813-1.

## Supplementary information

Supplementary Information Supplementary Methods, Discussion, Table 1 and References.

Reporting Summary

## Source data

Source Data Fig. 1 Replicate measurements of axon dimensions.

Source Data Fig. 2 Replicate measurements of axon dimensions.

Source Data Fig. 3 Replicate measurements of axon dimensions.

Source Data Fig. 4 Replicate measurements of axon dimensions.

Source Data Fig. 5 Replicate measurements of axon dimensions.

Source Data Extended Data Fig. 1 Replicate measurements of axon dimensions.

Source Data Extended Data Fig. 2 Replicate measurements of axon dimensions.

Source Data Extended Data Fig. 4 Replicate measurements of axon dimensions.

Source Data Extended Data Fig. 5 Replicate measurements of axon dimensions.

Source Data Extended Data Fig. 5 Unprocessed western blot in Extended Data Fig. 5a.

Source Data Extended Data Fig. 6 Replicate measurements of axon dimensions.

Source Data Extended Data Fig. 7 Replicate measurements of axon dimensions.

Source Data Extended Data Fig. 8 Replicate measurements of axon dimensions.

## Data Availability

Data are available upon request, and all correspondence should be addressed to P.R. and S.W. Source data are provided with this paper.

## Code availability

We used the software Mem3DG version 0.0.6rc2 to compute membrane shape change (https://github.com/RangamaniLabUCSD/Mem3DG). The AP modeling software that was used was Comsol Multiphysics version 6.1. The data analysis code is also available in GitHub at https://github.com/RangamaniLabUCSD/2023-axon-pearling/blob/main/.gitignore and Zenodo at 10.5281/zenodo.8060744 (ref. ^95^). All original code for EM, STED and electrophysiology analysis is archived on Zenodo at 10.5281/zenodo.8060707 (ref. ^96^) and is available from GitHub at https://github.com/RangamaniLabUCSD/2023-axon-pearling or upon request. Code from the laboratory of S.W. is available at https://github.com/shigekiwatanabe/axon_pearl_manuscript.
