# Supplementary material for: Membrane mechanics dictate axonal pearls-on-a-string morphology and function
Source: Nat Neurosci. 2024 Dec 2;28(1):49–61. doi: 10.1038/s41593-024-01813-1 (PMC11706780; doi:10.1038/s41593-024-01813-1)
Supplement: Supplementary file 2 — Reporting Summary [file 41593_2024_1813_MOESM2_ESM.pdf]

nature portfolio

Corresponding author(s): Padmini Rangamani and Shigeki Watanabe

Last updated by author(s): Aug 26, 2024

Reporting Summary

Nature Portfolio wishes to improve the reproducibility of the work that we publish. This form provides structure for consistency and transparency in reporting. For further information on Nature Portfolio policies, see our Editorial Policies and the Editorial Policy Checklist.

Statistics

For all statistical analyses, confirm that the following items are present in the figure legend, table legend, main text, or Methods section.

n/a	Confirmed	
☐	☒	The exact sample size (*n*) for each experimental group/condition, given as a discrete number and unit of measurement
☐	☒	A statement on whether measurements were taken from distinct samples or whether the same sample was measured repeatedly
☐	☒	The statistical test(s) used AND whether they are one- or two-sided<br>*Only common tests should be described solely by name; describe more complex techniques in the Methods section.*
☐	☒	A description of all covariates tested
☐	☒	A description of any assumptions or corrections, such as tests of normality and adjustment for multiple comparisons
☐	☒	A full description of the statistical parameters including central tendency (e.g. means) or other basic estimates (e.g. regression coefficient) AND variation (e.g. standard deviation) or associated estimates of uncertainty (e.g. confidence intervals)
☐	☒	For null hypothesis testing, the test statistic (e.g. *F*, *t*, *r*) with confidence intervals, effect sizes, degrees of freedom and *P* value noted<br>*Give P values as exact values whenever suitable.*
☒	☐	For Bayesian analysis, information on the choice of priors and Markov chain Monte Carlo settings
☒	☐	For hierarchical and complex designs, identification of the appropriate level for tests and full reporting of outcomes
☒	☐	Estimates of effect sizes (e.g. Cohen's *d*, Pearson's *r*), indicating how they were calculated

Our web collection on statistics for biologists contains articles on many of the points above.

Software and code

Policy information about availability of computer code

Data collection	For electron micrographs a custom code was created in Fiji Version 1.54e to annotate and measure NSVs and connectors. For STED imaging Imspector Image Acquisition & Analysis Software v16.3 was used.
Data analysis	For electron micrograph and STED analysis a custom code was created in MatLab R2021a to organize measurements and create a single output table. Graph Pad Prism 10.0.0 was used to create all graphs and perform all statistical analysis. All original codes are archived on Zenodo and available from Github or upon request. https://github.com/RangamaniLabUCSD/2023-axon-pearling and archived at http://doi.org/10.5281/zenodo.8060707. The codes from the Watanabe laboratory is available at https://github.com/shigekiwatanabe/axon_pearl_manuscript.

For manuscripts utilizing custom algorithms or software that are central to the research but not yet described in published literature, software must be made available to editors and reviewers. We strongly encourage code deposition in a community repository (e.g. GitHub). See the Nature Portfolio guidelines for submitting code & software for further information.

Data

Policy information about availability of data

All manuscripts must include a data availability statement. This statement should provide the following information, where applicable:

- Accession codes, unique identifiers, or web links for publicly available datasets
- A description of any restrictions on data availability
- For clinical datasets or third party data, please ensure that the statement adheres to our policy

All original data are available through Figshare https://figshare.com/account/home#/projects/170610 or upon request. All original codes are archived on Zenodo and available from Github or upon request. https://github.com/RangamaniLabUCSD/2023-axon-pearling and archived at http://doi.org/10.5281/zenodo.8060707.

Research involving human participants, their data, or biological material

Policy information about studies with human participants or human data. See also policy information about sex, gender (identity/presentation), and sexual orientation and race, ethnicity and racism.

Reporting on sex and gender	n/a
Reporting on race, ethnicity, or other socially relevant groupings	n/a
Population characteristics	n/a
Recruitment	n/a
Ethics oversight	n/a

Note that full information on the approval of the study protocol must also be provided in the manuscript.

Field-specific reporting

Please select the one below that is the best fit for your research. If you are not sure, read the appropriate sections before making your selection.

☒ Life sciences ☐ Behavioural & social sciences ☐ Ecological, evolutionary & environmental sciences

For a reference copy of the document with all sections, see nature.com/documents/nr-reporting-summary-flat.pdf

Life sciences study design

All studies must disclose on these points even when the disclosure is negative.

Sample size	Sample sizes are based on our previous experiments where they were sufficient for statistical analysis. Ref: Watanabe, S. et al. Ultrafast endocytosis at mouse hippocampal synapses. Nature 504, 242–247 (2013).<br>1. Watanabe, S. et al. Ultrafast endocytosis at Caenorhabditis elegans neuromuscular junctions. eLife 2013, (2013).<br>2. Watanabe, S. et al. Clathrin regenerates synaptic vesicles from endosomes. Nature 515, 228–233 (2014).<br>3. Kusick, G. F. et al. Synaptic vesicles transiently dock to refill release sites. Nature Neuroscience 23, 1329–1338 (2020).<br>4. Li, S. et al. Asynchronous release sites align with NMDA receptors in mouse hippocampal synapses. Nature Communications 2021 12:1 12, 1–13 (2021).
Data exclusions	Only one collection of data was excluded. It was an elecrophisiology recording from an acute slice where many of the neurons in the slice were going though apoptosis during the final recording.
Replication	All experimental conditions but the LatrunculinA treatment for 1 hr were done multiple times to verify reproducibility. The reason the LatrunclinA 1 hr treatment was only done once was because we saw that the 30 min treatment was better for neuron health and so this time point was used for further replicates. All attempts ad replication were successful.
Randomization	Samples were randomly assigned treatments.
Blinding	Investigators were blinded during allocation, sample preparation, sample processing, and data analysis. Only once all quantification was done were the investigators unblinded.

Reporting for specific materials, systems and methods

We require information from authors about some types of materials, experimental systems and methods used in many studies. Here, indicate whether each material, system or method listed is relevant to your study. If you are not sure if a list item applies to your research, read the appropriate section before selecting a response.

Materials & experimental systems

n/a	Involved in the study
☐	☒ Antibodies
☐	☒ Eukaryotic cell lines
☒	☐ Palaeontology and archaeology
☐	☒ Animals and other organisms
☒	☐ Clinical data
☒	☐ Dual use research of concern
☒	☐ Plants

Methods

n/a	Involved in the study
☒	☐ ChIP-seq
☒	☐ Flow cytometry
☒	☐ MRI-based neuroimaging

Antibodies

Antibodies used	anti-GAPDH antibody, Abcam, ab37168, polyclonal<br>anti-β-actin, SYSY, 251003, polyclonal<br>anti-ßII spectrin, Bd Cell Analysis, BDB612563, clone 42/B-Spectrin II<br>Goat anti-mouse IgG, Li-COR IRDye® 800 cw, 925-32210, polyclonal, Lot C90408-07<br>Goat anti-Rabbit IgG, Li-COR IRDye® 680RD (H + L), 925-68071, polyclonal, Lot D00115-05<br>anti-synaptophysin, SYSY, 101011, clone 7.2<br>anti-Ankyrin G, SYSY, 386004, polyclonal, Lot 1-6
Validation	anti-GAPDH antibody: validation by manufacturer in knockout cell line<br>anti-β-actin: independently validated by Science Exchange initiative<br>anti-ßII spectrin: validation by manufacturer by Western Blot "Western blot (Routinely Tested), Immunofluorescence (Tested During Development)"<br>Goat anti-mouse IgG: validation by manufacturer: "This antibody was tested by dot blot and/or solid-phase adsorbed for minimal cross-reactivity with human, rabbit, goat, rat, and horse serum proteins..."<br>Goat anti-Rabbit IgG: validation by manufacturer: "This antibody was tested by dot blot and/or solid-phase adsorbed for minimal cross-reactivity with human, mouse, rat, sheep, and chicken serum proteins..."<br>anti-synaptophysin: knock-out validated PubMed: 31940485<br>anti-Ankyrin G, kock-down validated PubMed: 31727776

Eukaryotic cell lines

Policy information about cell lines and Sex and Gender in Research

Cell line source(s)	HEK-293T cells, ATCC CRL-3216
Authentication	authentication by ATCC short tandem repeat (STR) profiling
Mycoplasma contamination	Not detected for mycoplasma contamination
Commonly misidentified lines<br>(See ICLAC register)	no commonly misidentified cell lines were used in the study

Animals and other research organisms

Policy information about studies involving animals; ARRIVE guidelines recommended for reporting animal research, and Sex and Gender in Research

Laboratory animals	Mus musculus, C57/BL6J, E18, P5-8, 8 weeks
Wild animals	The study did not involve wild animals
Reporting on sex	Sex was not considered in this study, both males and females were indistinguishably used in this study.
Field-collected samples	This study did not use any field-collected samples.
Ethics oversight	Animal Care and Use Committee at Johns Hopkins University School of Medicine<br>The European Union and CNRS UMR 5297 institutional guidelines for the care and use of laboratory animals (Council directive 2010/63/EU) and approved by the Committee of Ethics of Bordeaux (no. 50120198-A)<br>Swiss Federal Veterinary Office (the experimentation license 1889.3) All animals were housed with temperature control at 22 °C, 12 hr light/dark cycles,and ad libitum access to food and water.

Note that full information on the approval of the study protocol must also be provided in the manuscript.

Plants

Seed stocks	n/a
Novel plant genotypes	n/a
Authentication	n/a
