# Supplementary material for: Membrane mechanics dictate axonal pearls-on-a-string morphology and function
Source: Nat Neurosci. 2024 Dec 2;28(1):49–61. doi: 10.1038/s41593-024-01813-1 (PMC11706780; doi:10.1038/s41593-024-01813-1)

Raw western blot images:

Blot 1: Lane 1 – ladder, lane 2 – empty, lane 3 – βII knock down shRNA, lane 4 - βII knock down shRNA with 0.1% DMSO, lane 5 – scramble shRNA, lane 6 - untreated.

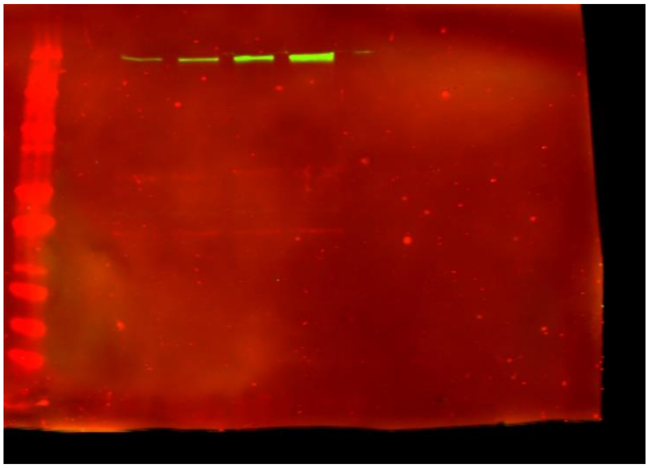

Blot 2: Lane 1 – ladder,
from one set of cultures: lane 2 – untreated, lane 3 – blebbistatin, lane 4 – nocodazole, lane 5 – scramble shRNA, lane 6 - scramble shRNA with LatA, lane 7 - βII knock down with LatA, lane 8 - βII knock down, lane 9 - βII knock down with 0.1% DMSO
from second set of cultures: lane 10 – untreated, lane 11 - scramble shRNA, lane 12 - βII knock down shRNA.
Note: only quantification for lanes 2, 5, 8, 10, 11, 12 are shown and relevent to this study.

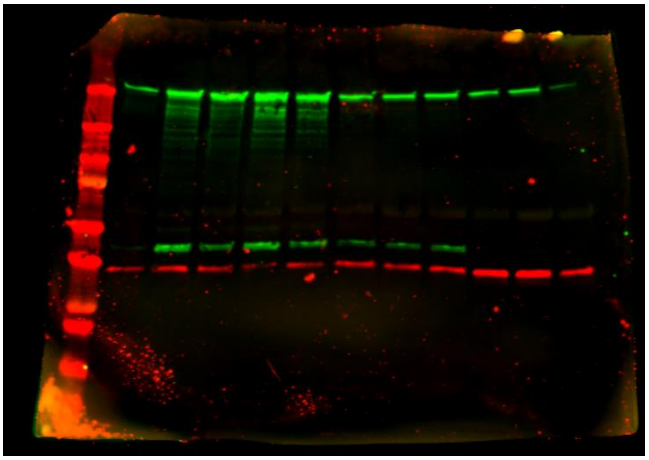

Supplement: Supplementary file 12 — Unprocessed western blot in Extended Data Fig. 5a. [file 41593_2024_1813_MOESM12_ESM.pdf]
